# Supplementary material for: Defective heart chamber growth and myofibrillogenesis after knockout of adprhl1 gene function by targeted disruption of the ancestral catalytic active site
Source: PLoS One. 2020 Jul 29;15(7):e0235433. doi: 10.1371/journal.pone.0235433 (PMC7390403; doi:10.1371/journal.pone.0235433)

S1.

Xenopus laevis adprhl1 transcripts and expression detected by exon-specific probes

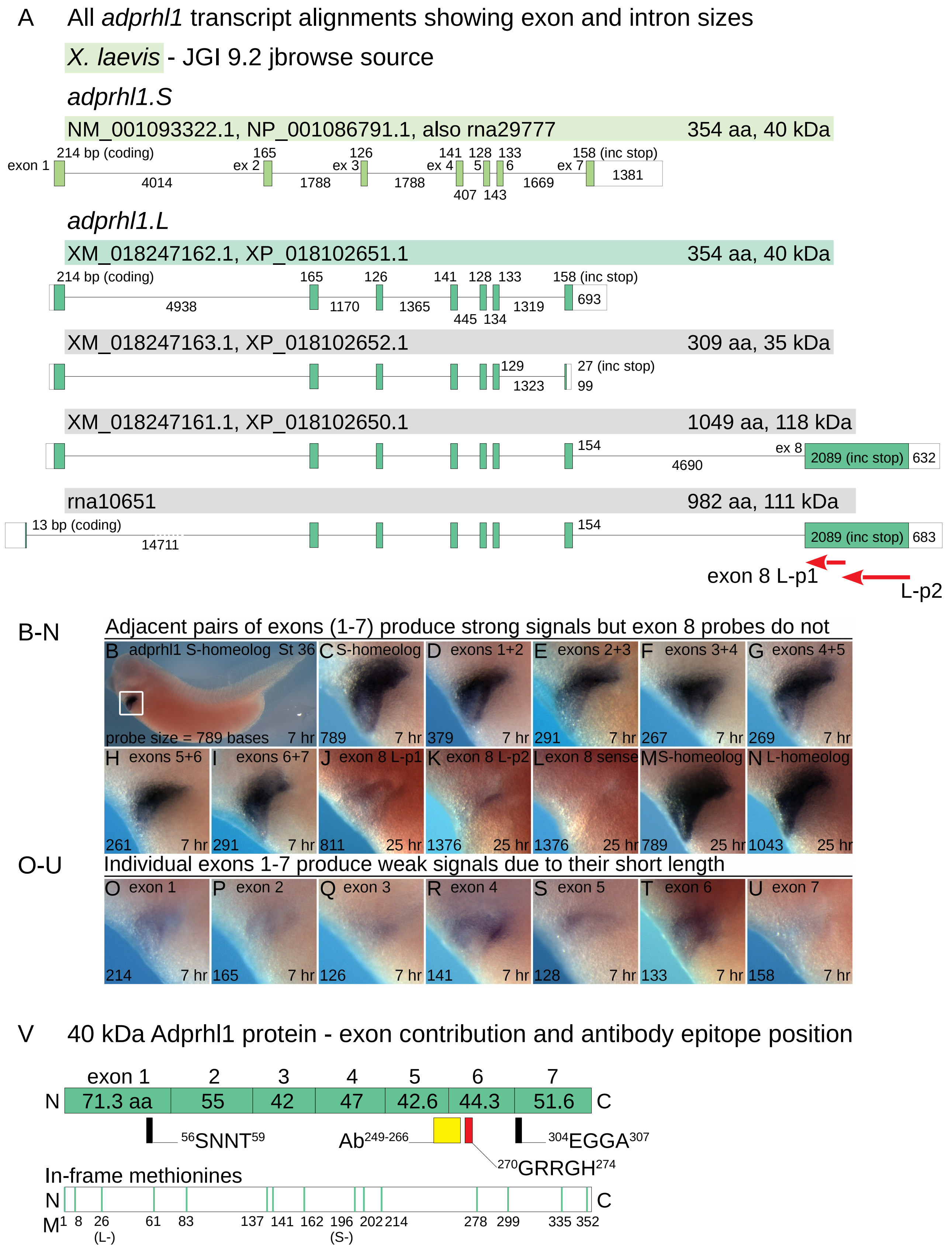

Supplement: S1 Fig — A: All X. laevis adprhl1 transcript alignments showing exon and intron sizes [26]. A single RefSeq mRNA aligns to the S-locus (NM_001093322.1, plus rna29777 that includes longer untranslated regions) while the L-locus is represented by four predicted transcripts. The L-transcript (XM_018247162.1) that closely matches the S-allele was cloned from cardiac cDNA and its sequence verified. Both have been used as hybridization probes in situ (B, C, M, N). The core coding sequence, comprising exons 1 through to 7, encodes a 40 kDa Adprhl1 protein. The precise composition of the prevalent 23 kDa Adprhl1 species also found in Xenopus hearts is unknown, although some progress mapping smaller proteins in mouse is presented (Results 3.11, Fig 8, Discussion 4.3). Current vertebrate gene alignments of adprhl1 include predicted longer transcripts containing an additional 3’-exon 8, which has been assigned to the X. laevis L-allele (XM_018247161.1, plus rna10651). Red arrows mark the position of two distinct exon 8 hybridization probes. The translated sequence from the L-p1 region is conserved with mammals whereas L-p2 is divergent. B-N: Adjacent pairs of exons (1–7) produce strong signals but exon 8 probes do not. A stage 36 tadpole (left-lateral view, B) and detail images of hearts (C-N) showing adprhl1 mRNA expression detected with region-specific antisense probes. The probe size (lower left, bases) and substrate incubation time (right, 7 or 25 hours) for the colour reaction is listed on each panel. Probes covering most of the coding sequence synthesized from either S- or L-locus cDNAs will detect the combined expression from all alleles (B, C, M, N). Smaller S-allele probes that correspond to pairs of adjacent exons between 1–7 (D-I) each produce strong heart signals equivalent to that observed using the larger 789 base coding fragment (B, C). In contrast to exons 1–7, the two L-p1 and L-p2 probes detect little cardiac expression from the putative exon 8, despite prolonged subs [file pone.0235433.s001.pdf]
