# Supplementary figures and images for: Defective heart chamber growth and myofibrillogenesis after knockout of adprhl1 gene function by targeted disruption of the ancestral catalytic active site
Source: PLoS One. 2020 Jul 29;15(7):e0235433. doi: 10.1371/journal.pone.0235433 (PMC7390403; doi:10.1371/journal.pone.0235433)

S2.

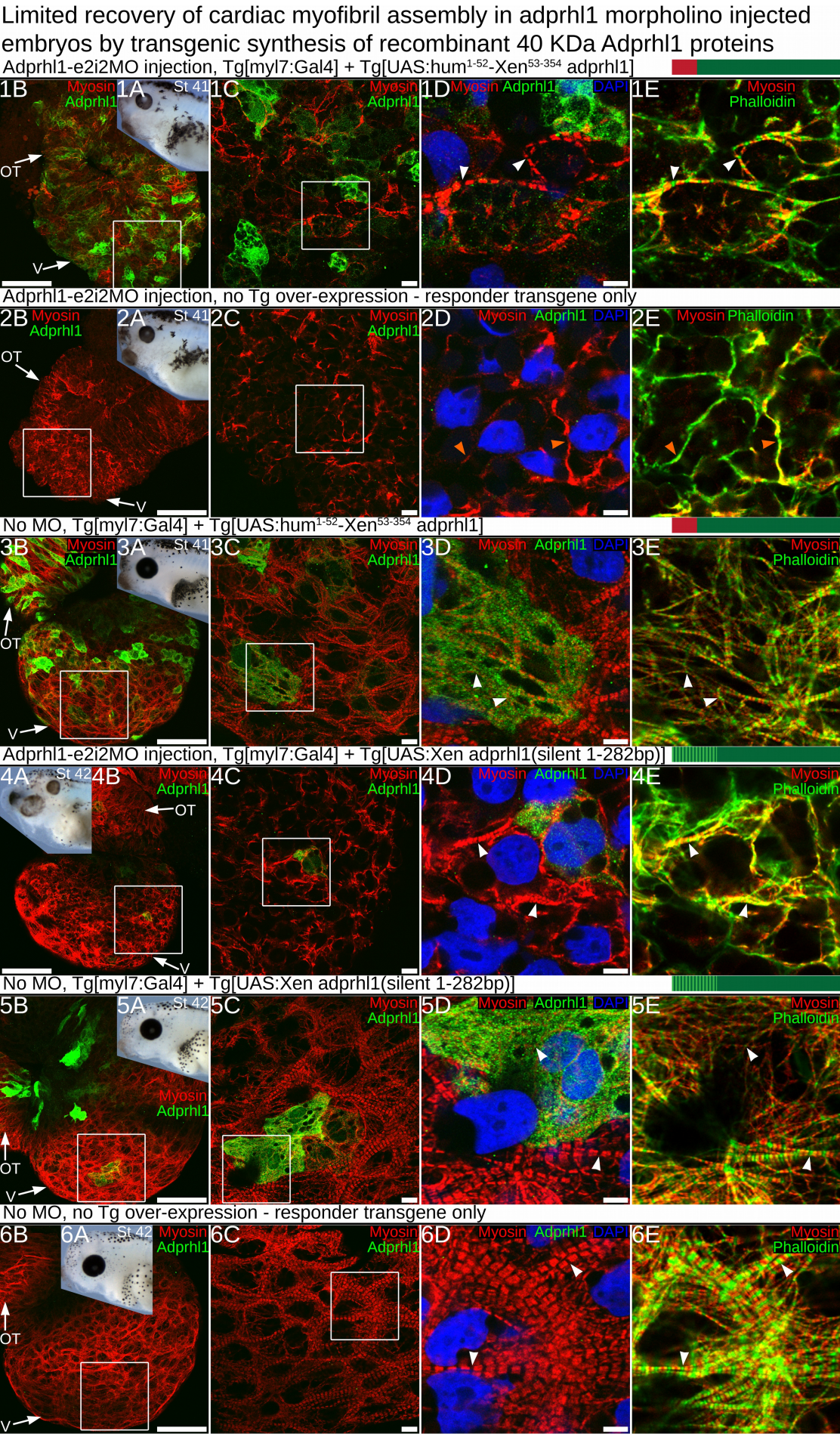

Supplement: S2 Fig — Experiments that combine adprhl1 MO knockdown with two distinct transgenes engineered to achieve adprhl1 over-expression. This is the extended version of Fig 2. It additionally shows the morphology of the experimental heart ventricles and the extent of Adprhl1 protein production within. The extra panels locate the position within each ventricle wall of the high magnification images (that are also presented in Fig 2) that reveal myofibril patterns found in sample cardiomyocytes. 1A-E: A stage 41 tadpole and its dissected heart ventricle that was injected with the RNA-splice interfering MO, Adprhl1-e2i2MO, into dorsal (D-2/4) blastomeres. Additionally, it carried binary transgenes to over-express recombinant Adprhl1 protein, consisting of Tg[myl7:Gal4] driver and the Tg[UAS:human1-52-Xenopus53-354 adprhl1] responder. Left lateral view of head and trunk (A), while the dissected heart was placed with the anterior surface uppermost (B). The white square (B) denotes the position of a detail image of the ventricle (C) and the white square (C) in turn marks the position of further magnified images (D, E). Scale bars = 100 μm (B), = 10 μm (C) and = 5 μm (D, E). Fluorescence images (B-D) show anti-Adprhl1 immunocytochemistry (green), anti-myosin (red) and DAPI-stained nuclei (blue, D). The final panel (E) displays a merge of myosin and phalloidin actin stain, with the phalloidin coloured green to evaluate signal overlap. 2A-E: A sibling tadpole that received the same Adprhl1-e2i2MO injection but carried only the UAS-responder transgene and hence did not produce excess recombinant Adprhl1. 3A-E: A double transgenic sibling that synthesized recombinant human-Xenopus hybrid Adprhl1 but was not injected with the MO. 4A-E: From a second experiment, a stage 42 tadpole that was injected with Adprhl1-e2i2MO and carried the Tg[myl7:Gal4] driver but a different Tg[UAS:Xenopus adprhl1(silent 1-282bp)] responder transgene. This incorporates silent nucleotide changes (synonymous substitut [file pone.0235433.s002.pdf]

## S5.

*Adprhl1* morpholinos - Position, sequence and activity in embryos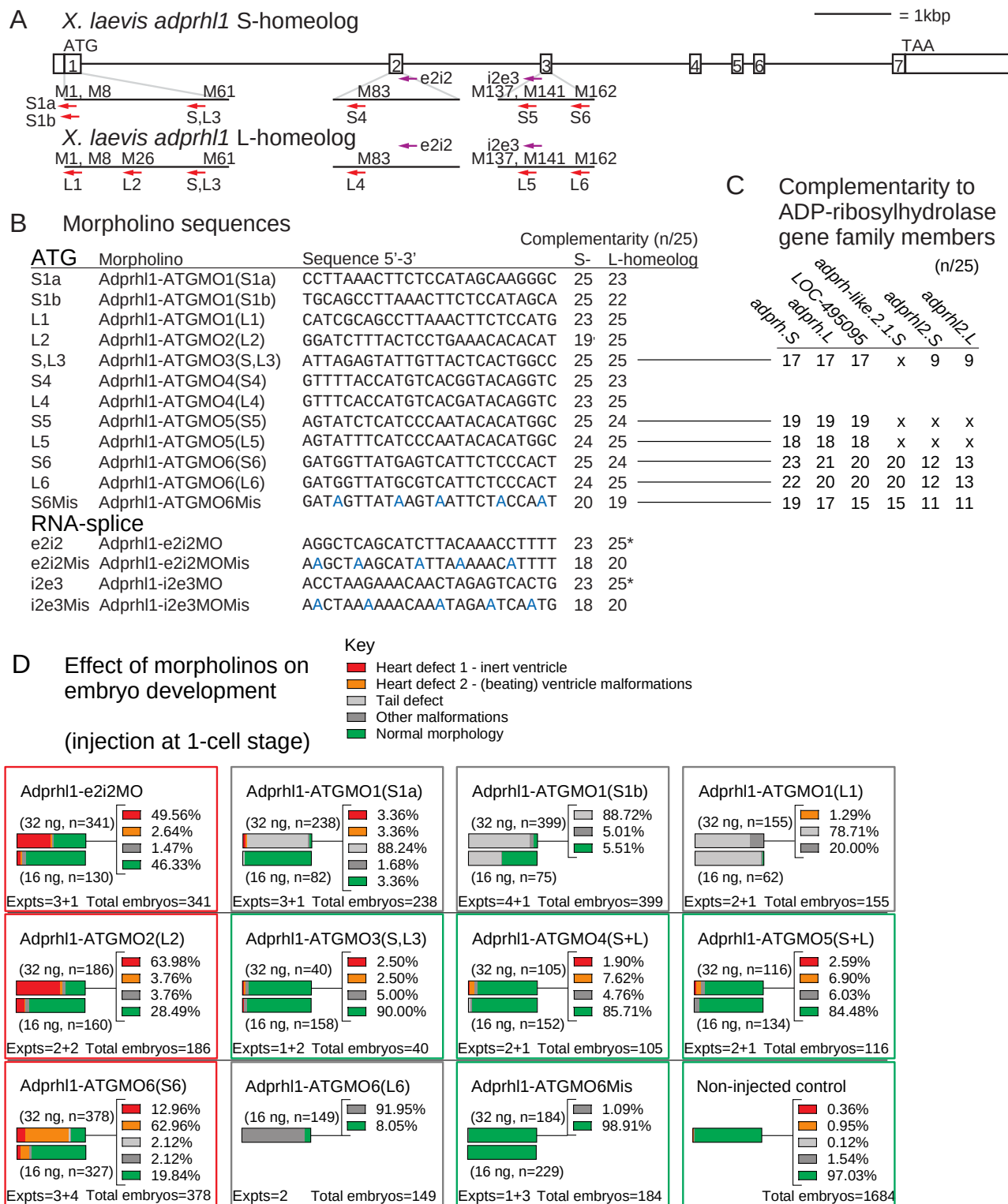

Supplement: S5 Fig — A: Diagram showing the hybridization position of MOs mapped to the first three exons of the S- and L-homeologous loci for X. laevis adprhl1. Morpholinos targeting potential translation initiation sites in adprhl1 mRNA are shown by red arrows, with the corresponding methionine from the Adprhl1 protein sequence printed above. In addition to the 5'-most ATG predicted as the start for 40 kDa translation, there are five internal ATG sequences within the same reading frame. Morpholinos that target RNA-splicing [10] are coloured violet. B: Table containing the MO sequences and their complementarity to the S- and L-homeologs of adprhl1. Asterisks (*) denote sequence variability within X. laevis (see Supplementary Methods 5.11 in S1 Data). Deliberately mismatched bases within control MOs are coloured blue. C: List showing the potential for hybridization to other members of the ADP-ribosylhydrolase gene family. Only MOs-S6 and L6 that target methionine-162 are noteworthy as they could cross-react with the four adprh loci that are present in Xenopus (adprh.S, adprh.L, LOC495095, adprh-like.2.1.S). D: The translation inhibition MOs produce varied effects on embryo development. Parts-of-whole charts showing the frequency of tadpole phenotypes assessed at stage 44 after 32 and 16 ng MO injection at the one-cell stage (percentage values listed for 32 ng injection). The number of independent experiments and total number of embryos assessed is given under each chart. Heart defects were observed for the MOs that interfere with adprhl1 RNA-splicing and for translation inhibiting MOs-L2 and S6. However, all three MOs designed to the most 5’-ATG (Met1), S1a, S1b and L1, caused unforeseen severe tail defects (after delayed blastopore closure at gastrulation). Morpholinos-3, 4 and 5 had no effect on embryo development. For MO-4 and 5, experiments are presented where a mixture of MOs targeting both S- and L-alleles was injected. Images showing representative embryos for active MOs are show [file pone.0235433.s005.pdf]

S7.

Activity of distinct Cas9 RNAs and protein for *tyrosinase* gene knockout in *X. laevis* embryos

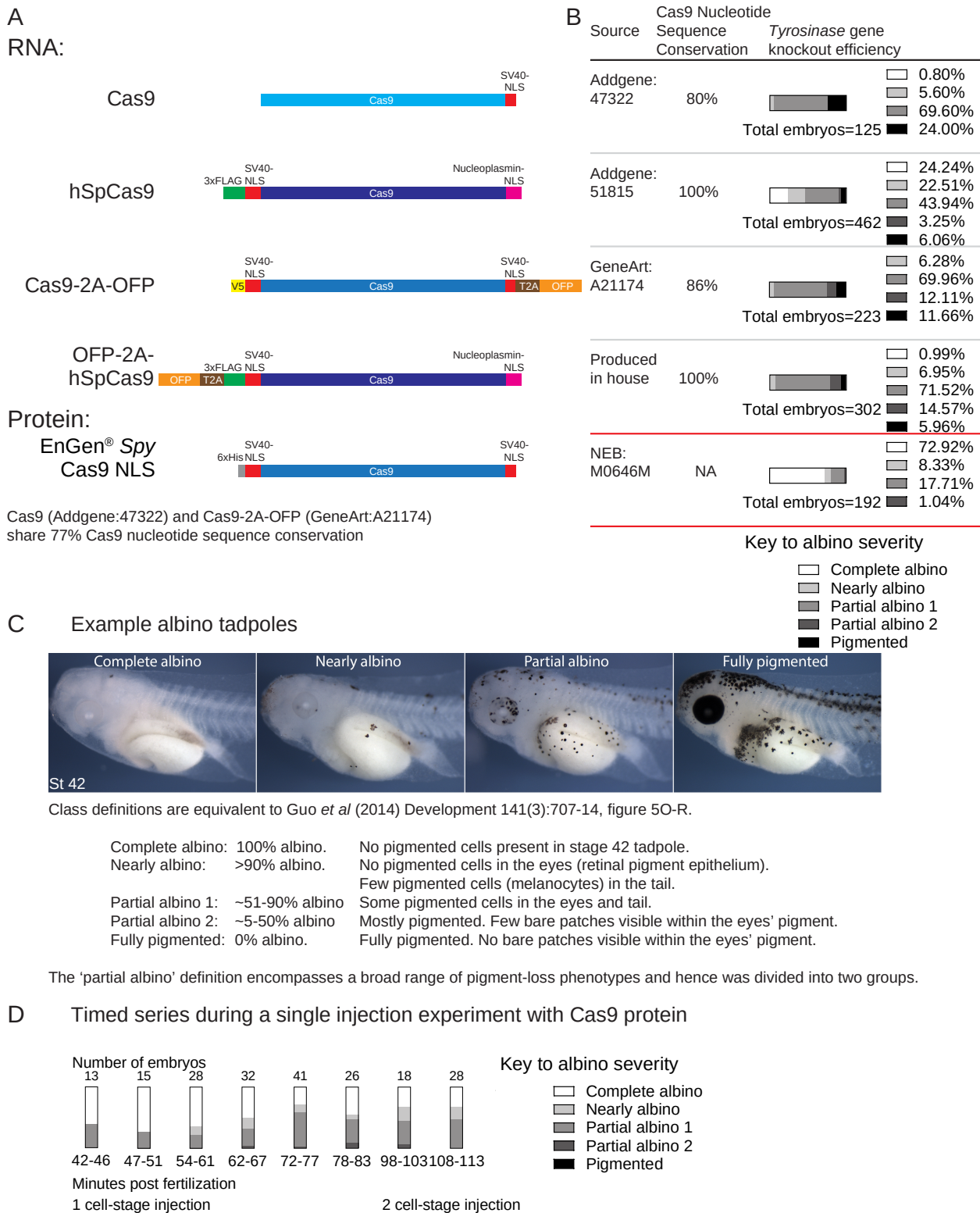

Supplement: S7 Fig — A: Comparing the coding sequence of four distinct Cas9 RNAs and the primary sequence of a commercial Cas9 protein preparation. The deduced Cas9 amino acid sequence originating from Streptococcus pyogenes is identical for all examples. Nevertheless, three shades of blue were used to depict Cas9 because the nucleotide sequences utilized differ due to distinct codon usage. Coding sequences were further modified by inclusion of epitope and purification tags (3xFLAG-green, V5-yellow, 6xHis-grey), nuclear localization signals (SV40-red, nucleoplasmin-magenta), self-cleaving 2A peptide (brown) and orange fluorescent protein reporter (orange). As an example of their size, the hSpCas9 protein is 1423 amino acids long, composed of N-terminal methionine, 22 aa 3xFLAG, 17 aa SV40-NLS, 1367 aa Cas9 (without N-term Met) and 16 aa nucleoplasmin-NLS sequences. B: Table showing the frequency of albino tadpoles obtained after disruption of the tyrosinase gene by injection of each Cas9 reagent together with tyr gRNAs into one-cell stage embryos (Materials and Methods 2.3). Parts-of-whole charts assigned the resulting stage 42 tadpoles to five albino phenotype classes that described the extent of pigmentation-loss and thus completeness of the gene knockout. C: Tadpoles representing the range of pigmentation-loss phenotypes observed after tyrosinase knockout. Left-lateral views, anterior half of tadpoles presented. Definitions of the five albino phenotype classes were comparable to those used by Guo et al [30]. D: Charts from a single experiment using Cas9 protein showing how the efficiency of tyrosinase knockout reduced as the time point of injection (minutes post-fertilization) increased. Beyond 90 minutes, embryos had reached the two-cell stage and thus injection of the same total mass of reagents was divided between both blastomeres. Based on this timed series, an upper limit of 60 minutes post-fertilization was set for all Cas9 injections at the one-cell stage. (PDF) [file pone.0235433.s007.pdf]

S16.

Mammalian *Adprhl1* transcript alignments don't predict exon 3-4 RNA-skipping

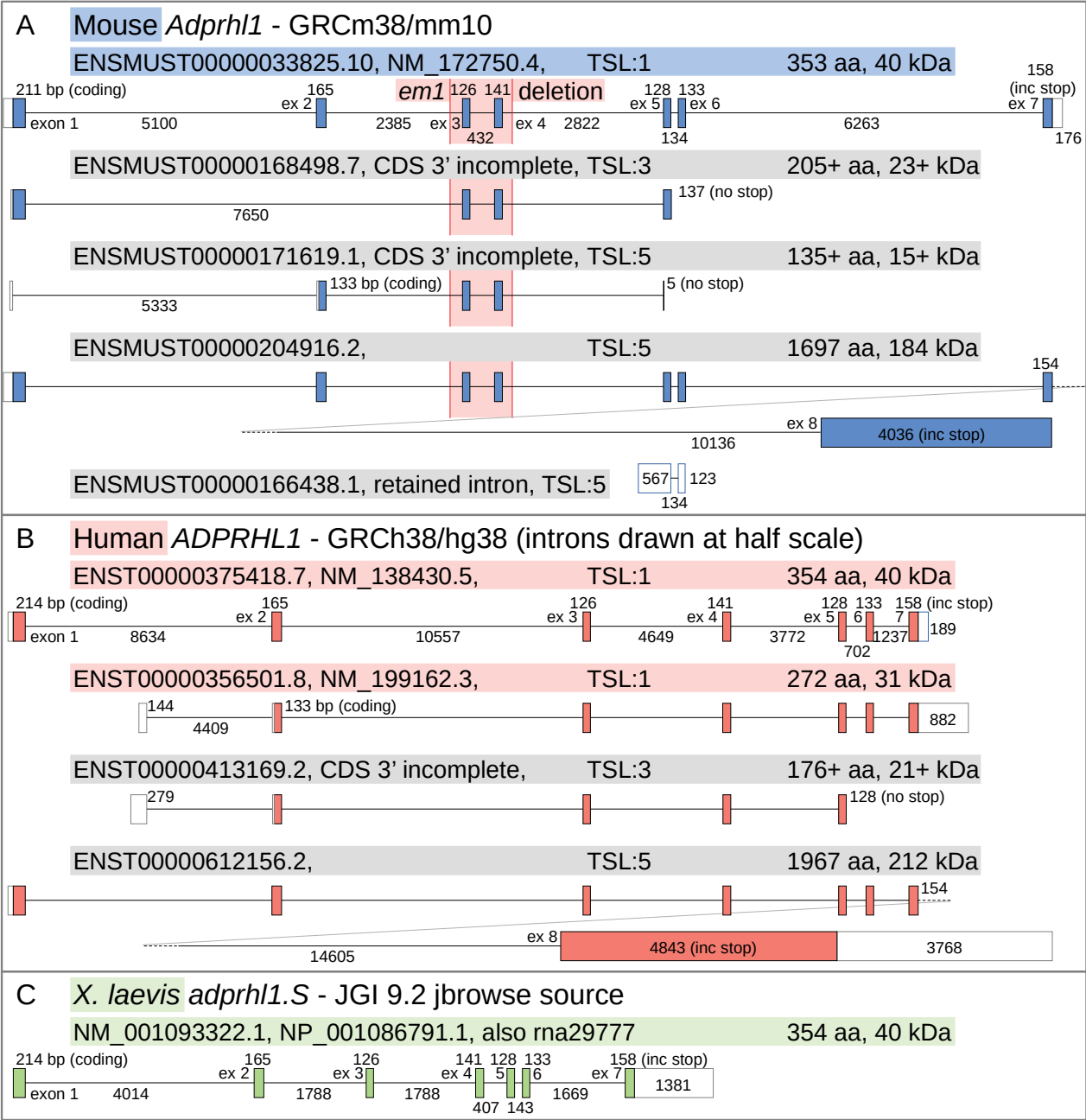

Supplement: S16 Fig — A: Five mouse Adprhl1 transcripts aligned to the gene showing exon and intron sizes. No mRNAs have yet been identified or predicted with exons 3 and 4 skipped in mouse, man or frog. The Transcript Support Level (TSL) values indicate low confidence for the four predicted mouse transcripts; one incorporates the additional 3’-exon 8, two have incomplete open reading frames and one is classed as a small non-coding retained intron. B: There are two RefSeq mRNAs arising from different promoters among the four human ADPRHL1 transcripts. C: For comparison, the X. laevis adprhl1 S-locus transcript alignment. (PDF) [file pone.0235433.s016.pdf]

**Fig 8 Original Images.**

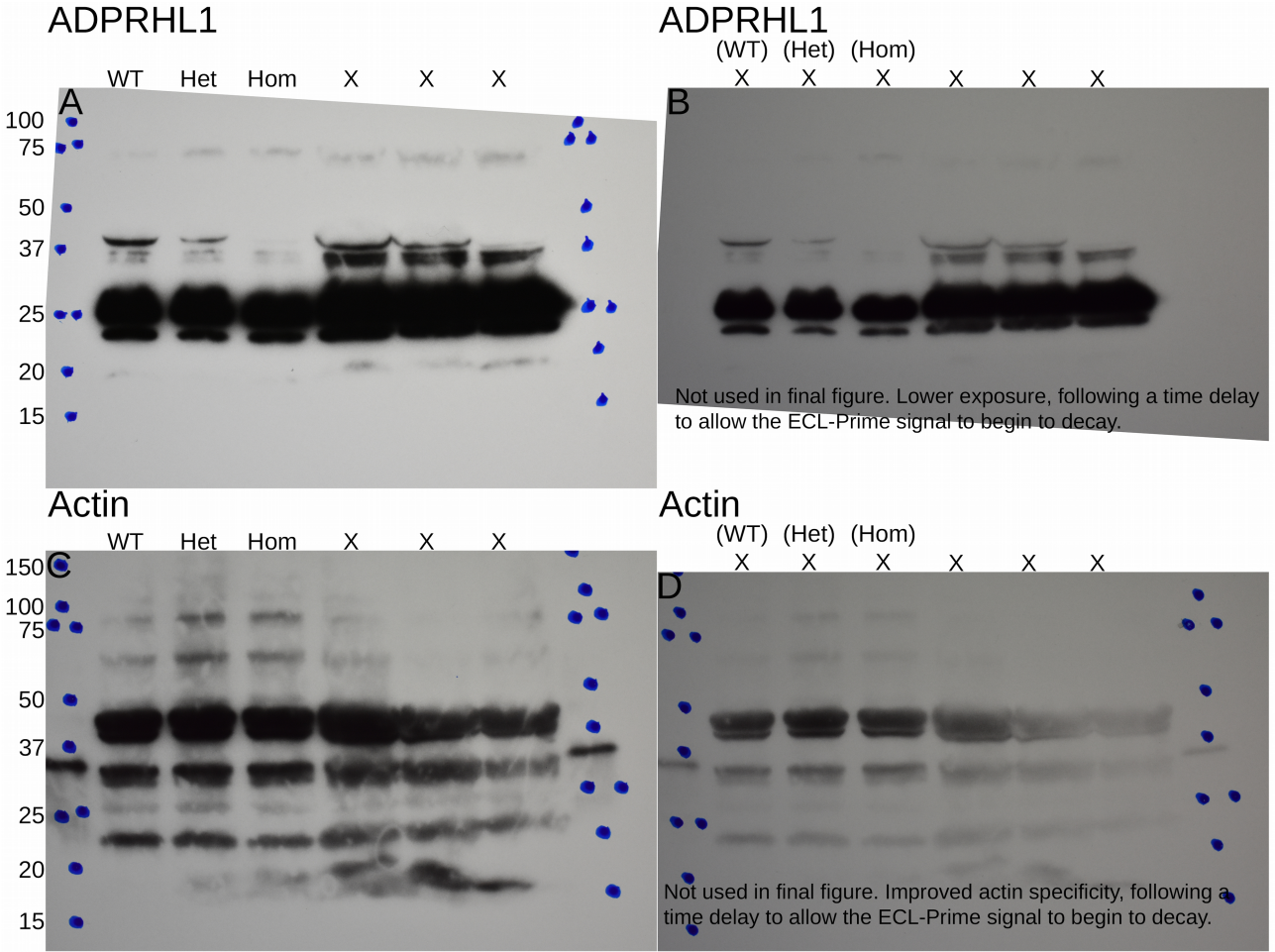

Supplement: S1 Raw image — Mice lacking Adprhl1 exons 3 and 4 are normal—They still produce 25 and 23 kDa ADPRHL1 proteins. A: ADPRHL1 protein from em1 allele mouse heart tissue. Left three lanes used for figure. Right three lanes contain twice the volume of extract loaded. Film exposure 10 seconds. B: Lower 5 seconds film exposure of the same experiment after a time delay to allow the ECL-Prime signal to begin to decay. C: Actin detection used to normalise loading of the samples. Left three lanes used for figure, higher loading of right three lanes has affected their resolution on the gel. Signal specificity not optimal due to excessive primary antibody concentration but the result serves to standardise the samples. Film exposure 4 seconds. D: Slightly improved specificity of the 42 kDa actin doublet following a longer time delay to allow the intense ECL-Prime signal to begin to decay. Film exposure 5 seconds. (PDF) [file pone.0235433.s017.pdf]

S3 Original Images.

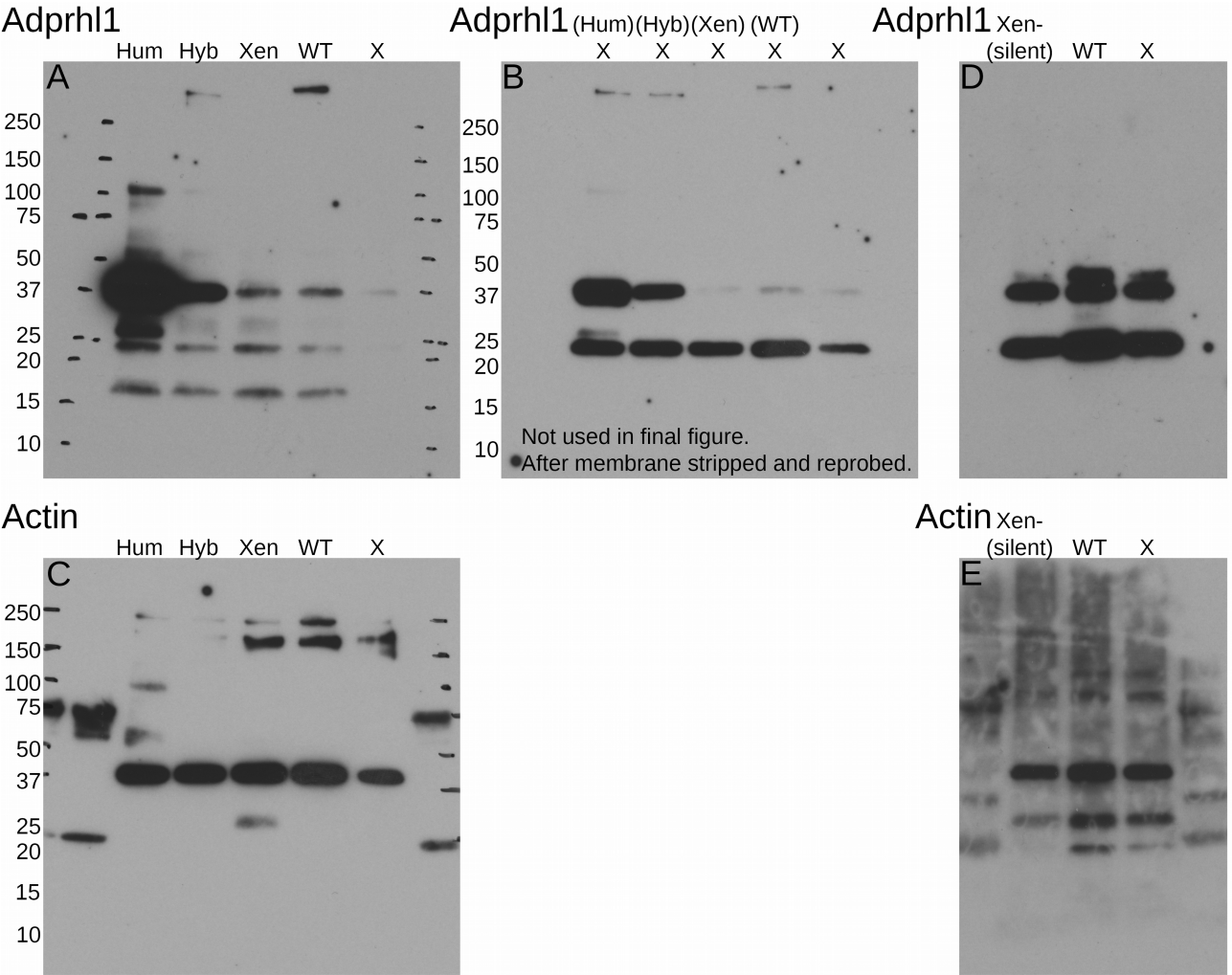

Supplement: S2 Raw image — Over-expression of recombinant 40 kDa Adprhl1 does not yield extra 23 kDa Adprhl1. The Adprhl1 (peptide) antibody gives a characteristic western blot signal pattern with Xenopus heart tissue. When used to probe freshly electrophoresed samples, the relative intensities of 40 and 23 kDa bands are usually comparable. Occasionally, an additional smaller 17 kDa product is also observed (A). If the membrane is then stripped for reuse with acidified glycine treatment, then any subsequent pattern obtained with the Adprhl1 antibody is subtly changed. The intensity of the 23 kDa signal is always increased relative to the 40 kDa band. No additional protein species are ever observed when used as a subsequent probe. Whether the 23 kDa Adprhl1 epitope is subject to (an obscuring) post-translational modification that can be removed by acid treatment remains to be explored. This feature of the Adprhl1 antibody is demonstrated here in panels A and B. Acid glycine treatment: freshly prepared 200 mM glycine, 1% SDS, pH 2.5; membrane washed twice for 10 minutes. A: Adprhl1 protein signal from different transgenic lines that was used in S3 Fig. Antibody probe of fresh samples. Film exposure 1 minute. B: Adprhl1 signal of the same samples obtained after the membrane was stripped and reprobed. This image was not used in S3 but provides useful insight into antibody activity. Film exposure 1 minute. C: Actin detection used to normalise loading of the samples. Film exposure 30 seconds. D: Adprhl1 signal from additional (fresh) samples, including from the transgenic line containing silent nucleotide changes. Film exposure 45 minutes. E: Actin signal to normalise loading of these additional samples. Film exposure 5 seconds. (PDF) [file pone.0235433.s018.pdf]
