# Supplementary material for: Defective heart chamber growth and myofibrillogenesis after knockout of adprhl1 gene function by targeted disruption of the ancestral catalytic active site
Source: PLoS One. 2020 Jul 29;15(7):e0235433. doi: 10.1371/journal.pone.0235433 (PMC7390403; doi:10.1371/journal.pone.0235433)

S3.

Over-expression of recombinant 40 kDa Adprhl1 does not yield extra 23 kDa Adprhl1

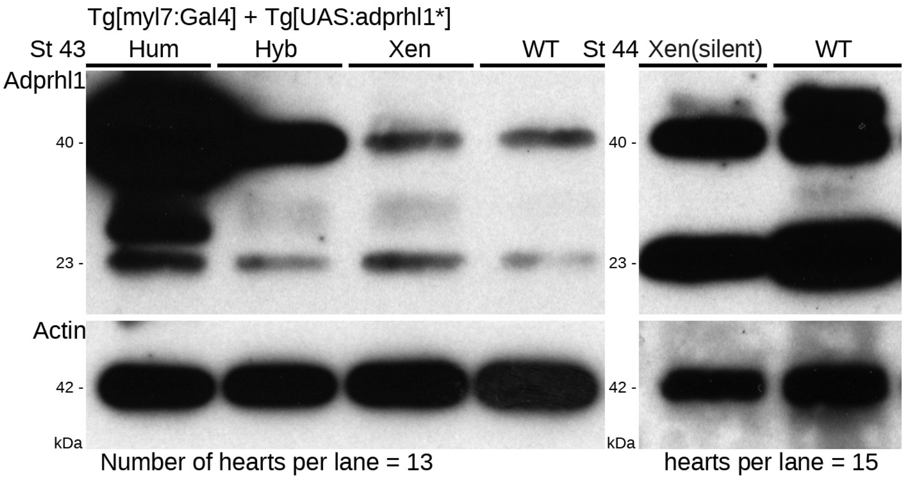

Supplement: S3 Fig — Western blots of transgenic tadpole hearts that carried stable lines of the Tg[myl7:Gal4] driver and one of a series of Tg[UAS:adprhl1] responders designed to over-express variants of 40 kDa Adprhl1 protein. Stage 43–44 heart extracts were probed with Adprhl1 antibody, with Actin detection used to normalize the samples. Hum corresponds to a Tg[UAS:human ADPRHL1] responder that induces large-scale synthesis of human-species ADPRHL1 protein in tadpole hearts. This transgene cDNA sequence is sufficiently different from Xenopus to evade the endogenous translational control mechanism that normally limits the production of Adprhl1. Hyb corresponds to the Tg[UAS:hum1-52-Xen53-354 adprhl1] responder that over-synthesizes a human-Xenopus hybrid form of Adprhl1 that also escapes translational control. Xen denotes a Tg[UAS:Xenopus adprhl1] transgene. Using unmodified Xenopus adprhl1 cDNA, transgene mRNA transcription is activated but no additional recombinant protein accumulates [10]. WT equates to control (wild type) hearts. On a separate gel with a higher signal exposure, Xen(silent) denotes the Tg[UAS:Xenopus adprhl1(silent 1-282bp)] transgene containing silent nucleotide changes to the 5’-Xenopus cDNA. This transgene partially evaded endogenous control and recombinant Adprhl1 accumulated in a fraction of the cardiomyocytes up to stage 42. However, there is a technical barrier to performing western blot analysis of transgenic hearts at these early stages. The time required to identify double-positive embryos by the transgenes’ marker eye fluorescence and subsequent sample preparation in the numbers necessary to obtain a signal is prohibitive. The silent mutation transgene gave a transient protein induction [10] and no additional Adprhl1 was detected in this sample prepared from stage 44 hearts. In hearts that synthesized excess 40 kDa hybrid Adprhl1, there was no commensurate increase in the abundance of the 23 kDa protein detected by the Adprhl1 antibody. It suggests the 2 [file pone.0235433.s003.pdf]
