# Supplementary material for: Defective heart chamber growth and myofibrillogenesis after knockout of adprhl1 gene function by targeted disruption of the ancestral catalytic active site
Source: PLoS One. 2020 Jul 29;15(7):e0235433. doi: 10.1371/journal.pone.0235433 (PMC7390403; doi:10.1371/journal.pone.0235433)
Supplement: S4 Fig — Tadpole heart ventricles with transgenic over-expression of 40 kDa Adprhl1 protein combined with markers of cell division (A-D) and cell death (E-R). The cardiac Tg[myl7:Gal4] driver and Tg[UAS:Xenopus adprhl1(silent 1-282bp)] responder transgenes were utilized. A-D: Two hearts showing anti-Adprhl1 (green), mitosis marker anti-phospho-Histone H3 (red) and phalloidin actin stain (magenta). White squares (A, C) denote the position of detail images (B, D). Cells undergoing mitosis were readily detected within all regions of embryonic stage 40–41 hearts. There was no correlation between excess Adprhl1 production and cell division. Mitotic cells usually had no Adprhl1 signal (B) but occasionally did contain Adprhl1 protein (D). Note, a second P-H3-positive cell (A) near to the featured cell lay deeper within the myocardial wall so was not detected by the thin optical section of the high magnification image (B). Scale bars = 100 μm (A, C), = 10 μm (B, D). E-I: Heart showing anti-Adprhl1 (green, E, F, H), ApopTag® TUNEL reaction stain (red, G, I), phalloidin (magenta, E, F, H) and DAPI (blue, F, H, I). The white square on the heart (Inset, E) locates images within the ventricle (F, G). Similarly, the squares (F, G) mark the position of detail images (H, I). The ApopTag® stain detects fragmented DNA of dying cells, caused by either apoptosis or necrotic destruction. The images focus on a cardiomyocyte on the ventricle anterior surface with excessive accumulation of Adprhl1 and a round appearance. Nevertheless, no ApopTag® signal was observed for this cell (G, I) nor indeed any Adprhl1-positive cells screened across 11 transgenic hearts. Scale bars = 100 μm (E, N), = 10 μm (F, G, J, K, O, P), = 5 μm (H, I, L, M, Q, R). J-M: Programmed cell death within the heart is a rare event during ventricle chamber outgrowth stages. Comparable images of a ventricle from a sibling transgenic tadpole (L has DAPI only). To prove the TUNEL reaction worked in situ, towards the apex, a solitar [file pone.0235433.s004.pdf]

S4.

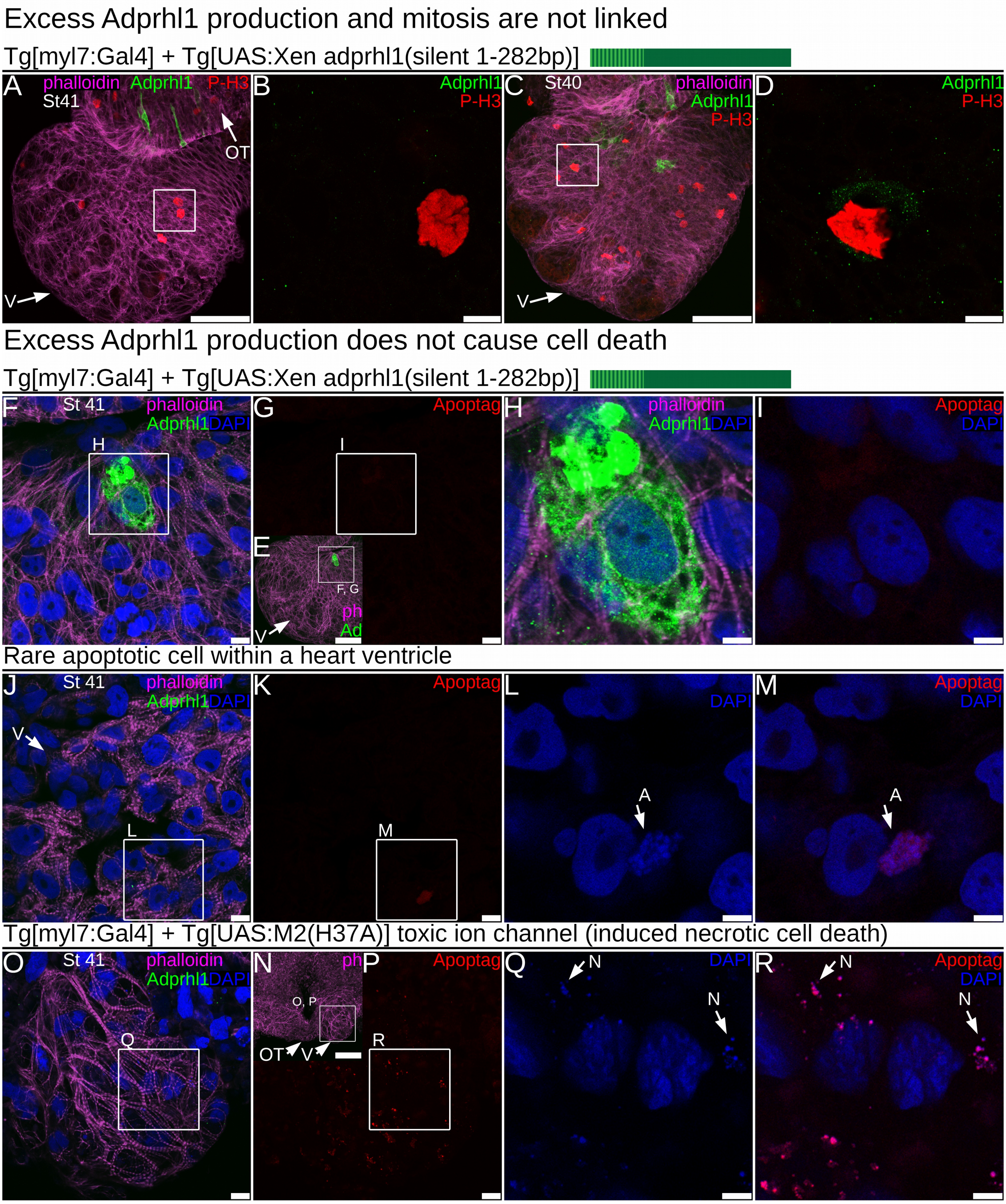

Excess Adprhl1 production and mitosis are not linked
Tg[myl7:Gal4] + Tg[UAS:Xen adprhl1(silent 1-282bp)]
A
phalloidin Adprhl1 P-H3
St41
OT
V
B
Adprhl1
P-H3
C
St40
phalloidin
Adprhl1
P-H3
V
D
Adprhl1
P-H3
Excess Adprhl1 production does not cause cell death
Tg[myl7:Gal4] + Tg[UAS:Xen adprhl1(silent 1-282bp)]
F
St 41
phalloidin
Adprhl1 DAPI
H
G
Apoptag
I
E
F, G
ph
Ad
V
H
phalloidin
Adprhl1 DAPI
I
Apoptag
DAPI
Rare apoptotic cell within a heart ventricle
J
St 41
phalloidin
Adprhl1 DAPI
V
L
K
Apoptag
M
L
DAPI
A
M
Apoptag
DAPI
A
Tg[myl7:Gal4] + Tg[UAS:M2(H37A)] toxic ion channel (induced necrotic cell death)
O
St 41
phalloidin
Adprhl1 DAPI
Q
N
ph
O, P
OT
V
P
Apoptag
R
Q
N
DAPI
N
R
N
Apoptag
DAPI
N
