# Supplementary material for: Defective heart chamber growth and myofibrillogenesis after knockout of adprhl1 gene function by targeted disruption of the ancestral catalytic active site
Source: PLoS One. 2020 Jul 29;15(7):e0235433. doi: 10.1371/journal.pone.0235433 (PMC7390403; doi:10.1371/journal.pone.0235433)
Supplement: S6 Fig — A: The diagram showing hybridization positions of MOs mapped to the first three exons of the S- and L-homeologous loci for X. laevis adprhl1. B, C: Reproduced for comparison, panels from Fig 1 featuring Adprhl1-e2i2MO RNA-splice interfering MO. Expression of actc1 (heart and skeletal muscle, B) and adprhl1 (C) mRNAs in stage 40 tadpoles after injection of 32 ng -e2i2MO. Impaired heart chamber growth and a loss of adprhl1 mRNA signal is observed. Left-lateral view of tadpole and detail ventral view of heart region presented. D, E: Normal ventricle size and adprhl1 signal in non-injected sibling tadpoles. F-O: Morpholinos designed to inhibit Adprhl1 protein translation produce varied effects on embryo development. Targeting distinct (but same reading frame) ATG-translation initiation sequences can yield malformations, but each exhibits a flaw that limits the MOs usefulness (see S5 Fig legend for details). F, G: Three overlapping MOs designed to the 5’-most AUG of adprhl1 mRNA each cause a tail growth defect, overshadowing any phenotype they might cause in the heart. Stage 39 tadpoles resulting from Adprhl1-ATGMO1(S1b) injection are shown. H-K: Adprhl1-ATGMO2(L2) injection results in inert hearts with small ventricles. Two severities of phenotype at stage 39 are shown, mildly affected tadpoles with small ventricles (H, J) and also details from tadpoles with a complete loss of ventricle growth (I, K). L, M: Adprhl1-ATGMO6(S6) gives some hearts that contract but have loss of chamber growth. N, O: Normal ventricle size and adprhl1 signal in non-injected stage 39 sibling tadpoles. Red arrows denote aberrant morphology. H, heart; T, tail. (PDF) [file pone.0235433.s006.pdf]

S6.

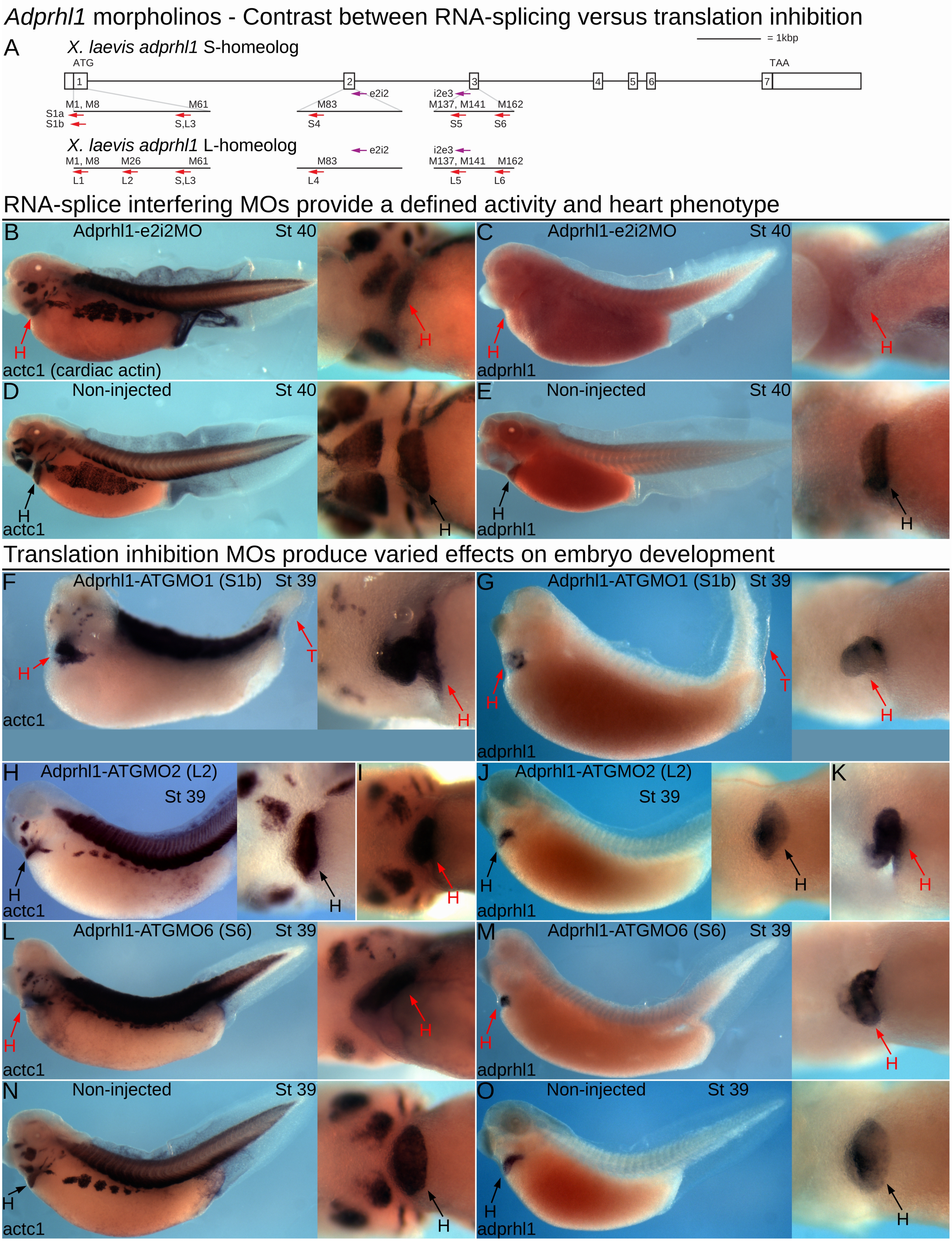

Adprhl1 morpholinos - Contrast between RNA-splicing versus translation inhibition
A
X. laevis adprhl1 S-homeolog
= 1kbp
ATG
TAA
1
2
3
4
5
6
7
e2i2
i2e3
M1, M8
M61
M83
M137, M141
M162
S1a
S1b
S,L3
S4
S5
S6
X. laevis adprhl1 L-homeolog
e2i2
i2e3
M1, M8
M26
M61
M83
M137, M141
M162
L1
L2
S,L3
L4
L5
L6
RNA-splice interfering MOs provide a defined activity and heart phenotype
B
Adprhl1-e2i2MO
St 40
H
actc1 (cardiac actin)
C
Adprhl1-e2i2MO
St 40
H
adprhl1
D
Non-injected
St 40
H
actc1
E
Non-injected
St 40
H
adprhl1
Translation inhibition MOs produce varied effects on embryo development
F
Adprhl1-ATGMO1 (S1b)
St 39
H
T
actc1
G
Adprhl1-ATGMO1 (S1b)
St 39
H
T
adprhl1
H
Adprhl1-ATGMO2 (L2)
St 39
actc1
I
J
Adprhl1-ATGMO2 (L2)
St 39
adprhl1
K
L
Adprhl1-ATGMO6 (S6)
St 39
actc1
M
Adprhl1-ATGMO6 (S6)
St 39
adprhl1
N
Non-injected
St 39
actc1
O
Non-injected
St 39
adprhl1
