# Supplementary material for: Defective heart chamber growth and myofibrillogenesis after knockout of adprhl1 gene function by targeted disruption of the ancestral catalytic active site
Source: PLoS One. 2020 Jul 29;15(7):e0235433. doi: 10.1371/journal.pone.0235433 (PMC7390403; doi:10.1371/journal.pone.0235433)

S8.

Adprhl1 gRNAs - Full list of gRNA experiments and embryo phenotype frequencies

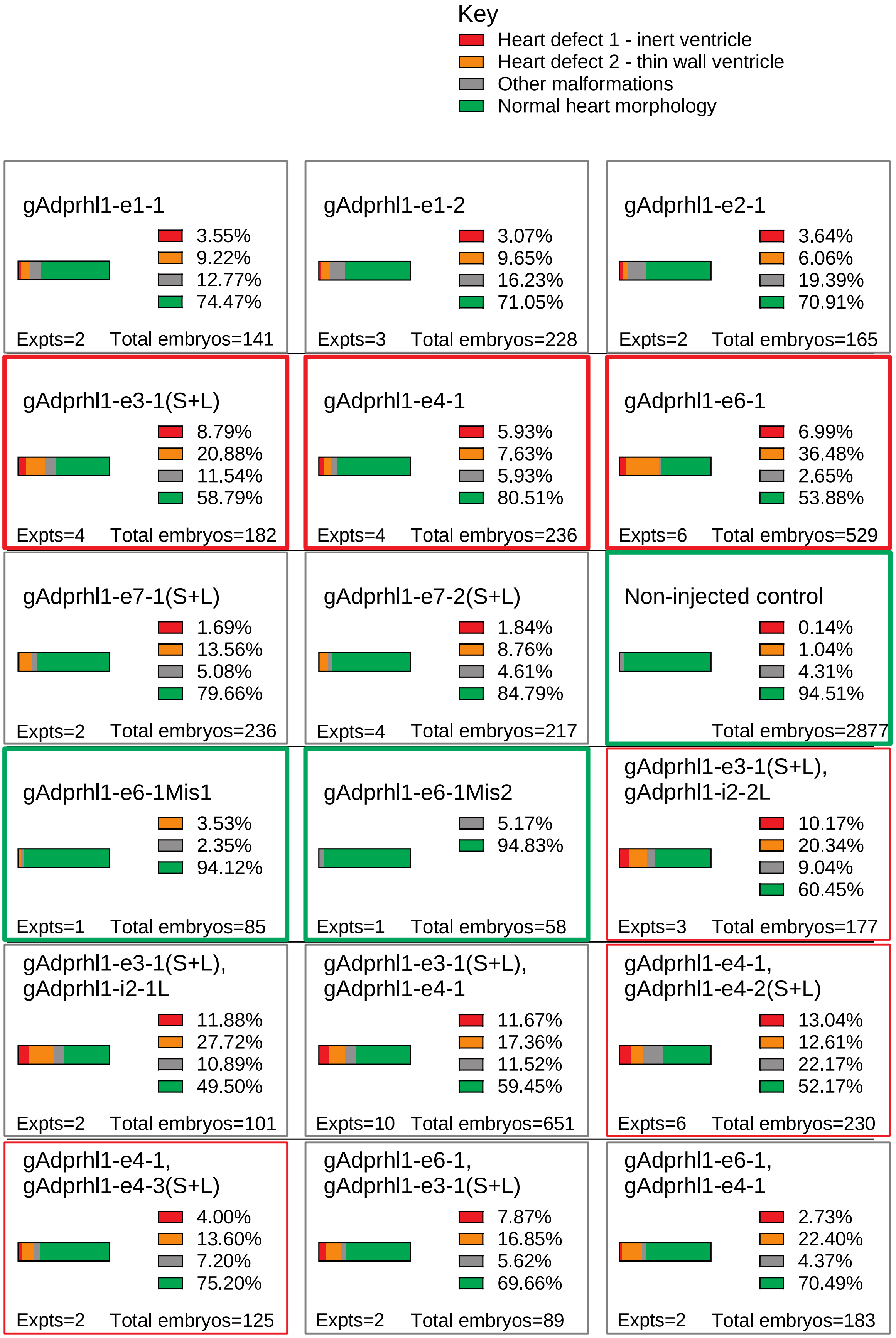

Supplement: S8 Fig — Parts-of-whole charts showing the frequency of stage 44 tadpole phenotypes that occurred after injection of adprhl1 gRNAs along with Cas9 protein into one-cell stage embryos. The number of independent experiments and total number of embryos assessed is given under each chart. Red rectangles surround graphs for gRNAs whose activities were also examined by DNA sequencing. Green rectangles denote control gRNAs and also a graph presenting the cumulative total for non-injected sibling tadpoles assayed in the experiments. The highest frequencies of heart defects were detected using the gAdprhl1-e3-1 and in particular the -e6-1 gRNA. The lower seven panels show the consequence of combining gRNAs that hybridize to two genomic regions of adprhl1 into a single injection. None of these combinatorial gRNA experiments increased heart defect frequencies beyond that obtained by gAdprhl1-e6-1 alone. Nonetheless, sequencing data was analysed for experiments where exons 3 and 4 were targeted at neighbouring positions to determine if resulting lesions contained deletions between the DSB sites (see S12 Fig). (PDF) [file pone.0235433.s008.pdf]
