# Supplementary material for: Defective heart chamber growth and myofibrillogenesis after knockout of adprhl1 gene function by targeted disruption of the ancestral catalytic active site
Source: PLoS One. 2020 Jul 29;15(7):e0235433. doi: 10.1371/journal.pone.0235433 (PMC7390403; doi:10.1371/journal.pone.0235433)
Supplement: S9 Fig — Sanger DNA sequences of adprhl1 S-homeologous locus exon 3 after mutation by the gAdprhl1-e3-1(S+L) gRNA plus Cas9. Mutated sequences from the L-locus gave the same profile. The hybridization position of the gRNA is depicted by the red arrow placed above the expected sequence (top 2 rows, exon and genomic). Alignment of 215 cloned (S-) isolates of amplified DNA obtained from 23 tadpoles, with every sequence carrying a lesion at the gRNA binding site. Mutant nucleotide sequences are coloured red. Missense mutations are listed first, followed by deletions (red hyphens, ordered by ascending size) and then sequences containing insertions (red arrowheads). Columns to the right give the number of instances of each sequence, alongside a genotype score that records the consequence of the given mutation to the Adprhl1 primary amino acid sequence. The key to interpret the genotype score is also included. (PDF) [file pone.0235433.s009.pdf]

S9.

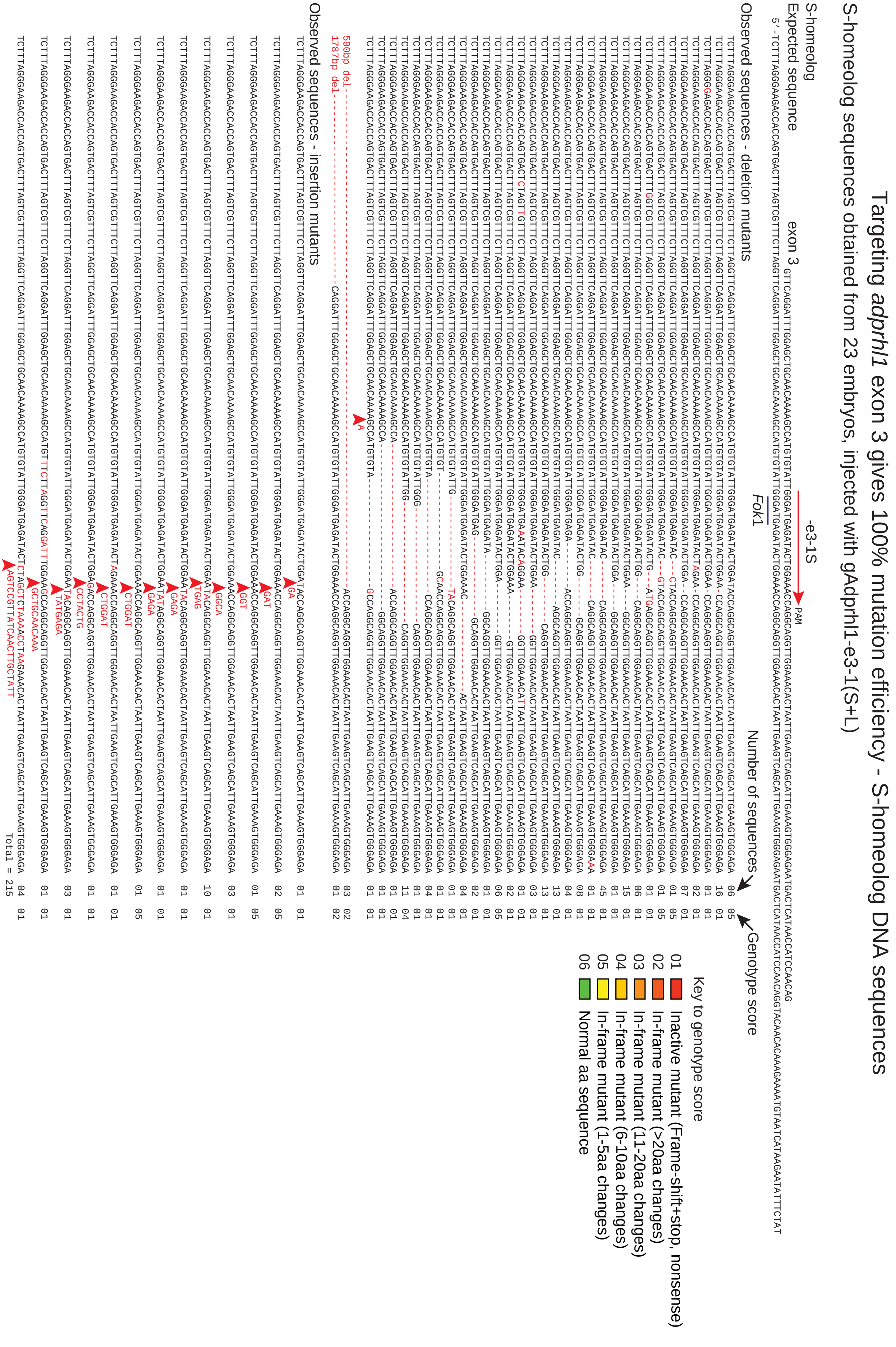
Targeting adprhl1 exon 3 gives 100% mutation efficiency - S-homeolog DNA sequences
S-homeolog sequences obtained from 23 embryos, injected with gAdprhl1-e3-1(S+L)
S-homeolog
Expected sequence
exon 3
-e3-1S
PAM
Fok1
Observed sequences - deletion mutants
Observed sequences - insertion mutants
590bp del
1787bp del
Number of sequences
Genotype score
Total = 215
Key to genotype score
01 Inactive mutant (Frame-shift+stop, nonsense)
02 In-frame mutant (>20aa changes)
03 In-frame mutant (11-20aa changes)
04 In-frame mutant (6-10aa changes)
05 In-frame mutant (1-5aa changes)
06 Normal aa sequence
