# Supplementary material for: Defective heart chamber growth and myofibrillogenesis after knockout of adprhl1 gene function by targeted disruption of the ancestral catalytic active site
Source: PLoS One. 2020 Jul 29;15(7):e0235433. doi: 10.1371/journal.pone.0235433 (PMC7390403; doi:10.1371/journal.pone.0235433)
Supplement: S10 Fig — A: All DNA sequences grouped by gRNAs injection. Each DNA sequence of adprhl1 exon 3 obtained after gRNA plus Cas9-mediated mutation was assigned a genotype score (a number code), based on the size of amino acid sequence modification it encoded. Parts-of-whole charts record the frequency of these genotype scores tallied for all embryos that received the -e3-1(S+L) gRNA (left chart), an embryo injected with a combination of neighbouring -e3-1(S+L) and -i2-2L gRNAs (centre chart), and a non-injected control embryo (right chart). The total number of S- and L-locus sequences analysed is listed below each chart. The key to interpret the genotype score is also included (far right). The inclusion of the -i2-2L gRNA induced larger deletions at the L-locus that would lead to exon 3 skipping, as shown by the increased proportion of score 02 sequences. B: Sequences and mutation details of individual embryos. Separate charts for 11 of the embryos, including 5 tadpoles that developed heart defects, 5 that developed normally and the one non-injected sibling. Columns list the number of sequences and the number of distinct sequences (in square brackets) for each embryo, plus the presence of larger deletions (and their size) that skip exon 3 and score 02. More importantly, for those embryos harbouring subtle mutations that scored 04 or 05, the precise amino acid change is also given. A standard nomenclature for protein sequence variations is used to describe the changes, with a simplified summary (highlighted) underneath stating whether the mutation caused a missense, deletion or aa insertion modification. Highlight colour matches the genotype score of the sequence. The rectangle border colour denotes a missense (teal), net deletion (blue) or net insertion (violet). At this position within exon 3, wherever missense mutations were found (eg p.(Lys145Ile)), the tadpole had preserved a normal heart morphology. C: Structure model of X. laevis Adprhl1 protein backbone, oriented with the [file pone.0235433.s010.pdf]

S10.

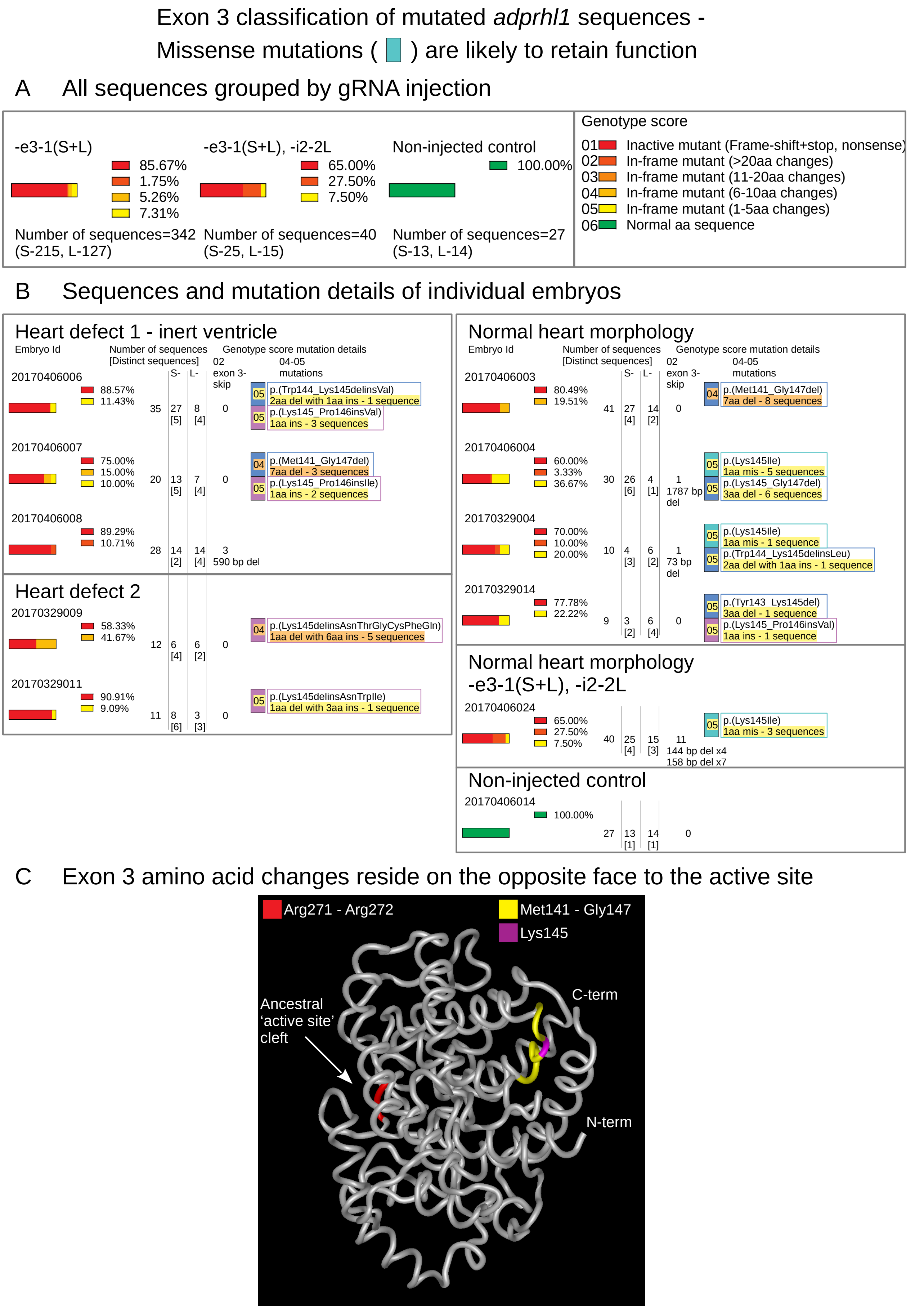
Exon 3 classification of mutated adprhl1 sequences -
Missense mutations ( ) are likely to retain function
A All sequences grouped by gRNA injection
-e3-1(S+L)
85.67%
1.75%
5.26%
7.31%
Number of sequences=342
(S-215, L-127)
-e3-1(S+L), -i2-2L
65.00%
27.50%
7.50%
Number of sequences=40
(S-25, L-15)
Non-injected control
100.00%
Number of sequences=27
(S-13, L-14)
Genotype score
01 Inactive mutant (Frame-shift+stop, nonsense)
02 In-frame mutant (>20aa changes)
03 In-frame mutant (11-20aa changes)
04 In-frame mutant (6-10aa changes)
05 In-frame mutant (1-5aa changes)
06 Normal aa sequence
B Sequences and mutation details of individual embryos
Heart defect 1 - inert ventricle
Embryo Id
Number of sequences [Distinct sequences]
S-
L-
Genotype score mutation details
02 exon 3-skip
04-05 mutations
20170406006
88.57%
11.43%
35 27 [5] 8 [4] 0
05 p.(Trp144_Lys145delinsVal) 2aa del with 1aa ins - 1 sequence
05 p.(Lys145_Pro146insVal) 1aa ins - 3 sequences
20170406007
75.00%
15.00%
10.00%
20 13 [5] 7 [4] 0
04 p.(Met141_Gly147del) 7aa del - 3 sequences
05 p.(Lys145_Pro146insIle) 1aa ins - 2 sequences
20170406008
89.29%
10.71%
28 14 [2] 14 [4] 3 590 bp del
Heart defect 2
20170329009
58.33%
41.67%
12 6 [4] 6 [2] 0
04 p.(Lys145delinsAsnThrGlyCysPheGln) 1aa del with 6aa ins - 5 sequences
20170329011
90.91%
9.09%
11 8 [6] 3 [3] 0
05 p.(Lys145delinsAsnTrpIle) 1aa del with 3aa ins - 1 sequence
Normal heart morphology
Embryo Id
Number of sequences [Distinct sequences]
S-
L-
Genotype score mutation details
02 exon 3-skip
04-05 mutations
20170406003
80.49%
19.51%
41 27 [4] 14 [2] 0
04 p.(Met141_Gly147del) 7aa del - 8 sequences
20170406004
60.00%
3.33%
36.67%
30 26 [6] 4 [1] 1 1787 bp del
05 p.(Lys145Ile) 1aa mis - 5 sequences
05 p.(Lys145_Gly147del) 3aa del - 6 sequences
20170329004
70.00%
10.00%
20.00%
10 4 [3] 6 [2] 1 73 bp del
05 p.(Lys145Ile) 1aa mis - 1 sequence
05 p.(Trp144_Lys145delinsLeu) 2aa del with 1aa ins - 1 sequence
20170329014
77.78%
22.22%
9 3 [2] 6 [4] 0
05 p.(Tyr143_Lys145del) 3aa del - 1 sequence
05 p.(Lys145_Pro146insVal) 1aa ins - 1 sequence
Normal heart morphology
-e3-1(S+L), -i2-2L
20170406024
65.00%
27.50%
7.50%
40 25 [4] 15 [3] 11 144 bp del x4 158 bp del x7
05 p.(Lys145Ile) 1aa mis - 3 sequences
Non-injected control
20170406014
100.00%
27 13 [1] 14 [1] 0
C Exon 3 amino acid changes reside on the opposite face to the active site
Arg271 - Arg272
Met141 - Gly147
Lys145
C-term
Ancestral 'active site' cleft
N-term
