# Supplementary material for: Defective heart chamber growth and myofibrillogenesis after knockout of adprhl1 gene function by targeted disruption of the ancestral catalytic active site
Source: PLoS One. 2020 Jul 29;15(7):e0235433. doi: 10.1371/journal.pone.0235433 (PMC7390403; doi:10.1371/journal.pone.0235433)
Supplement: S11 Fig — DNA sequences of adprhl1 exon 4 after mutation by the gAdprhl1-e4-1 gRNA plus Cas9, with both S-homeologous locus (upper panel) and L-locus (lower panel) presented. The hybridization position of -e4-1 gRNA is shown by the red arrow and neighbouring gRNAs by grey arrows, placed above the expected sequence (top 2 rows, exon and genomic). Alignment of 93 DNA clones (S-63, L-30) of amplified DNA obtained from 5 tadpoles. 76 sequences were mutated but 17 remained unaltered, demonstrating this gRNA was less effective than those that targeted exons 3 and 6. As before, mutant nucleotide sequences are coloured red. Wild-type (WT) sequences are listed first, followed by missense mutations, deletions (red hyphens) and insertions (red arrowheads). The number of instances of each sequence is to the right, alongside its genotype score. (PDF) [file pone.0235433.s011.pdf]

S11.

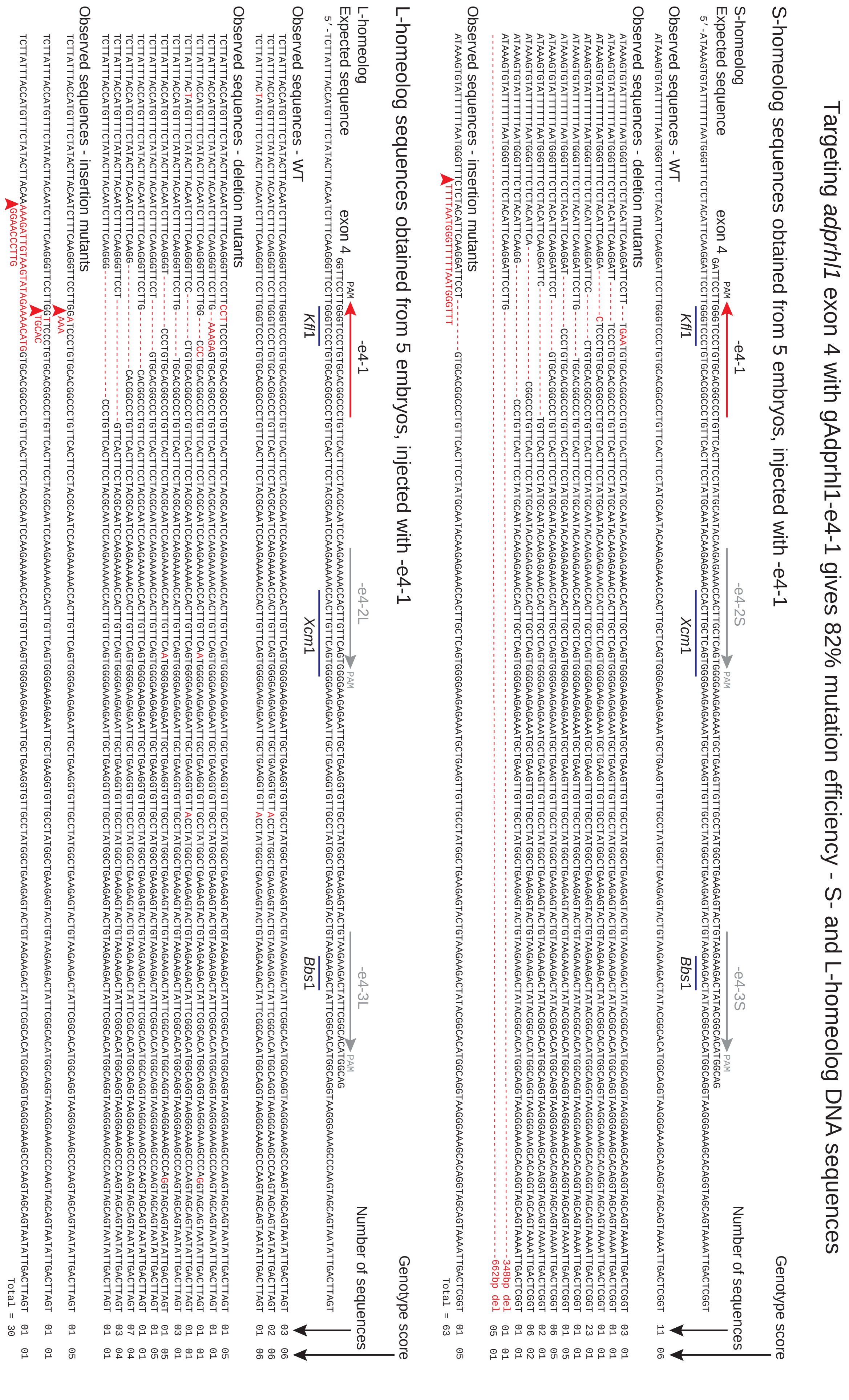
Targeting adprhl1 exon 4 with gAdprhl1-e4-1 gives 82% mutation efficiency - S- and L-homeolog DNA sequences
S-homeolog sequences obtained from 5 embryos, injected with -e4-1
S-homeolog
Expected sequence
Observed sequences - WT
Observed sequences - deletion mutants
Observed sequences - insertion mutants
Number of sequences
Genotype score
L-homeolog sequences obtained from 5 embryos, injected with -e4-1
L-homeolog
Expected sequence
Observed sequences - WT
Observed sequences - deletion mutants
Observed sequences - insertion mutants
Total = 63
Total = 30
