# Supplementary material for: Defective heart chamber growth and myofibrillogenesis after knockout of adprhl1 gene function by targeted disruption of the ancestral catalytic active site
Source: PLoS One. 2020 Jul 29;15(7):e0235433. doi: 10.1371/journal.pone.0235433 (PMC7390403; doi:10.1371/journal.pone.0235433)
Supplement: S13 Fig — A: All DNA sequences grouped by gRNA injection. Parts-of-whole charts record the frequency of sequence genotype scores tallied for all embryos that received Cas9 and gRNAs targeting adprhl1 exon 4. The gAdprhl1-e4-1 gRNA was injected individually (far-left chart) and in combination with the neighbouring -e4-2(S+L) (centre-left chart) or -e4-3(S+L) gRNAs (centre-right chart), while sequences from non-injected control embryos were also compared (far-right chart). The total number of S- and L-locus sequences analysed is listed below each chart. The key to interpret the genotype score is also included. Mutation at exon 4 was extensive but incomplete and some wild-type sequences persisted for all combinations of gRNAs tested. B: Sequences and mutation details of individual embryos. Separate charts for 8 typical embryos with normal heart formation, including two representing each gRNA mixture plus sibling controls. Columns list the number of sequences and the number of distinct sequences (in square brackets) for each embryo, plus the presence of larger deletions (and their size) that skip exon 4 and genotype score 02. For smaller in-frame lesions, the precise amino acid change is also given. For these attempts to disrupt exon 4, most tadpoles retained a small proportion of wild-type sequence alleles. Of two presented here with 100% mutation, the reason their hearts were unaffected could not be determined conclusively. Nonetheless, one featured a subtle missense substitution while the second contained a deletion of 3 amino acids that would shorten an α-helix. They were also both notable for harbouring alleles likely to skip exon 4 from the mature mRNA. C: Structure model of X. laevis Adprhl1 protein backbone, oriented with the ancestral active site cleft to the left and facing away (Arg271-Arg272 coloured red). Exon 4 encodes a whole number of codons, contributing 47 amino acids. The start and end points of exon 4-derived amino acid sequence lie in close proximity within t [file pone.0235433.s013.pdf]

S13.

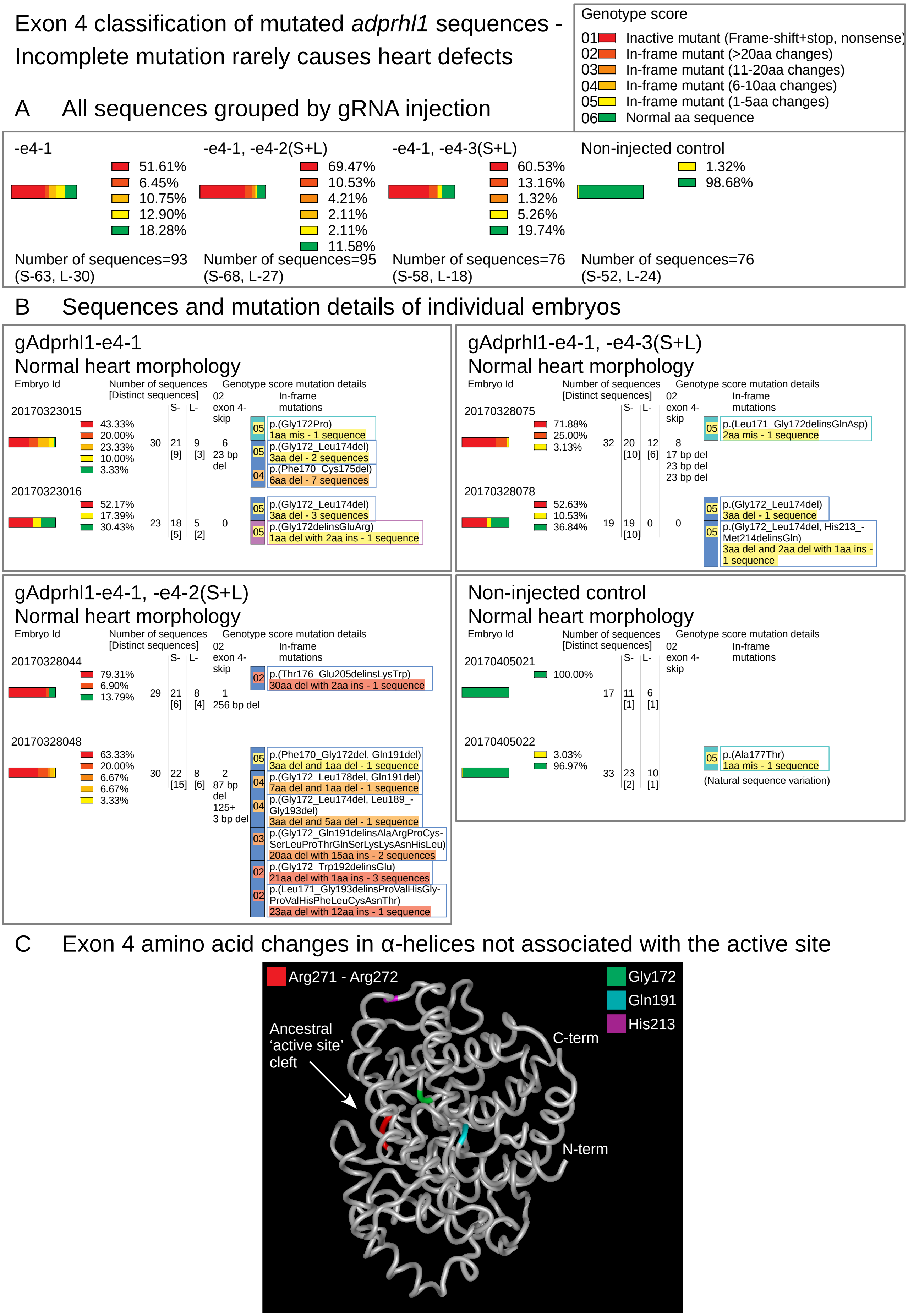
Exon 4 classification of mutated adprhl1 sequences -
Incomplete mutation rarely causes heart defects
Genotype score
01 Inactive mutant (Frame-shift+stop, nonsense)
02 In-frame mutant (>20aa changes)
03 In-frame mutant (11-20aa changes)
04 In-frame mutant (6-10aa changes)
05 In-frame mutant (1-5aa changes)
06 Normal aa sequence
A All sequences grouped by gRNA injection
-e4-1
51.61%
6.45%
10.75%
12.90%
18.28%
Number of sequences=93
(S-63, L-30)
-e4-1, -e4-2(S+L)
69.47%
10.53%
4.21%
2.11%
2.11%
11.58%
Number of sequences=95
(S-68, L-27)
-e4-1, -e4-3(S+L)
60.53%
13.16%
1.32%
5.26%
19.74%
Number of sequences=76
(S-58, L-18)
Non-injected control
1.32%
98.68%
Number of sequences=76
(S-52, L-24)
B Sequences and mutation details of individual embryos
gAdprhl1-e4-1
Normal heart morphology
Embryo Id
Number of sequences
[Distinct sequences]
S-
L-
Genotype score mutation details
02
exon 4-
skip
In-frame
mutations
20170323015
43.33%
20.00%
23.33%
10.00%
3.33%
30
21
[9]
9
[3]
6
23 bp
del
05 p.(Gly172Pro)
1aa mis - 1 sequence
05 p.(Gly172_Leu174del)
3aa del - 2 sequences
04 p.(Phe170_Cys175del)
6aa del - 7 sequences
20170323016
52.17%
17.39%
30.43%
23
18
[5]
5
[2]
0
05 p.(Gly172_Leu174del)
3aa del - 3 sequences
05 p.(Gly172delinsGluArg)
1aa del with 2aa ins - 1 sequence
gAdprhl1-e4-1, -e4-3(S+L)
Normal heart morphology
Embryo Id
Number of sequences
[Distinct sequences]
S-
L-
Genotype score mutation details
02
exon 4-
skip
In-frame
mutations
20170328075
71.88%
25.00%
3.13%
32
20
[10]
12
[6]
8
17 bp del
23 bp del
23 bp del
05 p.(Leu171_Gly172delinsGlnAsp)
2aa mis - 1 sequence
20170328078
52.63%
10.53%
36.84%
19
19
[10]
0
0
05 p.(Gly172_Leu174del)
3aa del - 1 sequence
05 p.(Gly172_Leu174del, His213_-
Met214delinsGln)
3aa del and 2aa del with 1aa ins -
1 sequence
gAdprhl1-e4-1, -e4-2(S+L)
Normal heart morphology
Embryo Id
Number of sequences
[Distinct sequences]
S-
L-
Genotype score mutation details
02
exon 4-
skip
In-frame
mutations
20170328044
79.31%
6.90%
13.79%
29
21
[6]
8
[4]
1
256 bp del
02 p.(Thr176_Glu205delinsLysTrp)
30aa del with 2aa ins - 1 sequence
20170328048
63.33%
20.00%
6.67%
6.67%
3.33%
30
22
[15]
8
[6]
2
87 bp
del
125+
3 bp del
05 p.(Phe170_Gly172del, Gln191del)
3aa del and 1aa del - 1 sequence
04 p.(Gly172_Leu178del, Gln191del)
7aa del and 1aa del - 1 sequence
04 p.(Gly172_Leu174del, Leu189_-
Gly193del)
3aa del and 5aa del - 1 sequence
03 p.(Gly172_Gln191delinsAlaArgProCys-
SerLeuProThrGlnSerLysLysAsnHisLeu)
20aa del with 15aa ins - 2 sequences
02 p.(Gly172_Trp192delinsGlu)
21aa del with 1aa ins - 3 sequences
02 p.(Leu171_Gly193delinsProValHisGly-
ProValHisPheLeuCysAsnThr)
23aa del with 12aa ins - 1 sequence
Non-injected control
Normal heart morphology
Embryo Id
Number of sequences
[Distinct sequences]
S-
L-
Genotype score mutation details
02
exon 4-
skip
In-frame
mutations
20170405021
100.00%
17
11
[1]
6
[1]
20170405022
3.03%
96.97%
33
23
[2]
10
[1]
05 p.(Ala177Thr)
1aa mis - 1 sequence
(Natural sequence variation)
C Exon 4 amino acid changes in α-helices not associated with the active site
Arg271 - Arg272
Gly172
Gln191
His213
Ancestral
'active site'
cleft
C-term
N-term
