# Supplementary material for: Defective heart chamber growth and myofibrillogenesis after knockout of adprhl1 gene function by targeted disruption of the ancestral catalytic active site
Source: PLoS One. 2020 Jul 29;15(7):e0235433. doi: 10.1371/journal.pone.0235433 (PMC7390403; doi:10.1371/journal.pone.0235433)
Supplement: S14 Fig — Cloned DNA sequences of adprhl1 L-locus exon 6 after mutation by the gAdprhl1-e6-1 gRNA plus Cas9. Mutated sequences from the S-locus exon 6 are presented in Fig 4. Alignment of 249 (L-) DNA clones obtained from 16 tadpoles, with 248 carrying a lesion and just one instance of a wild-type (WT) sequence persisting. Mutant nucleotide sequences are coloured red. Deletion mutations are listed first (red hyphens) and then insertions (red arrowheads). Frequently occurring sequences containing in-frame 3, 9 or 12 bp deletions are highlighted. The number of instances of each sequence is to the right, alongside its genotype score. Note, a handful of exon 6 clones had a mixed origin, containing S-sequence at one end and L-sequence at the other end of the PCR fragment. They must have been produced by two distinct PCR annealing and partial extension reactions occurring for a single DNA strand, exacerbated by the short extension times recommended by modern proof-reading DNA polymerases. They were assigned to S- or L- datasets according to the homeolog identity at the 5’- of the PCR. The first and nineteenth rows of this figure feature two such clones, classed as L-sequences but with 6 mismatches that have an S-origin. (PDF) [file pone.0235433.s014.pdf]

S14.

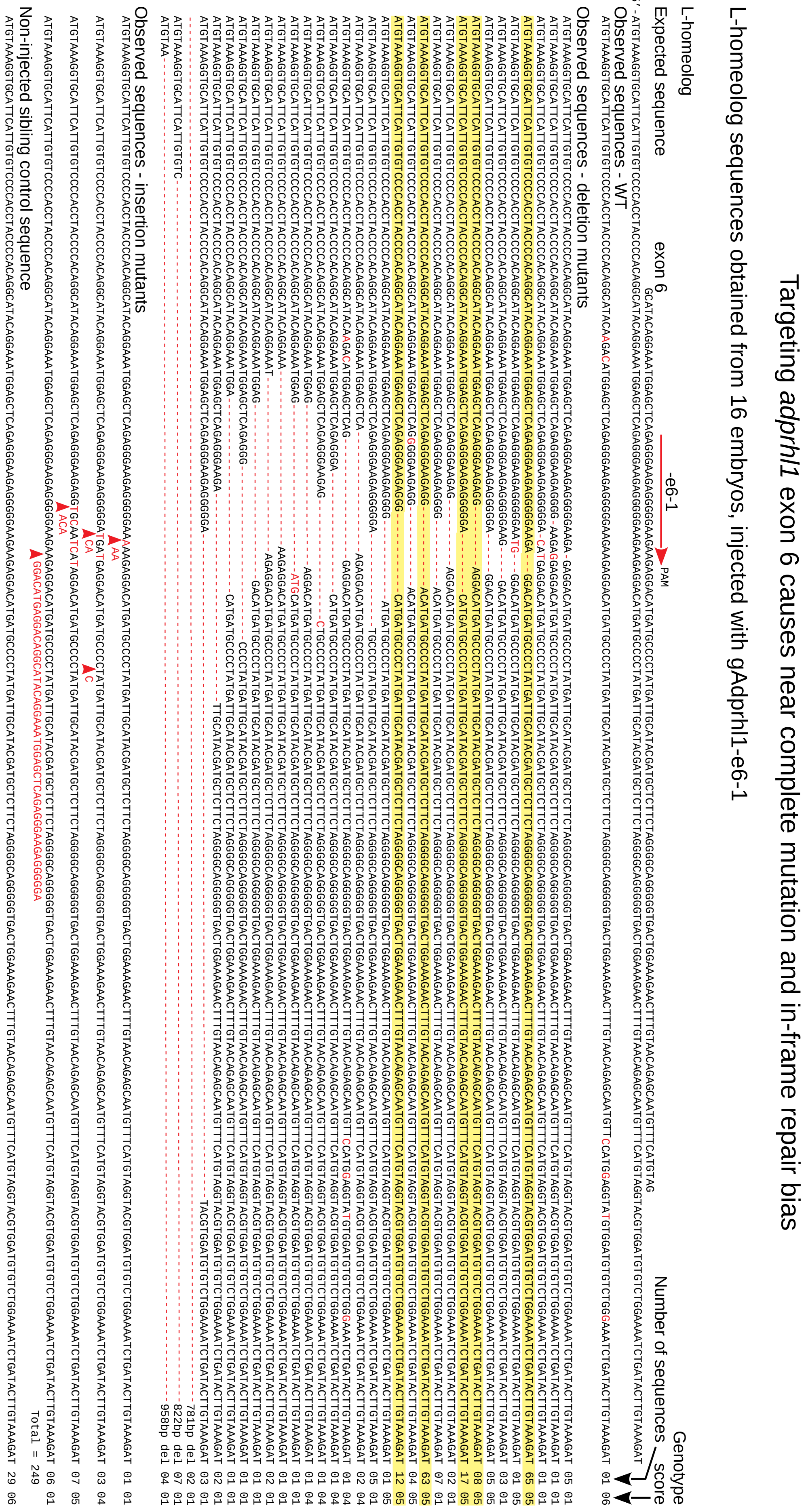
Targeting adprhl1 exon 6 causes near complete mutation and in-frame repair bias
L-homeolog sequences obtained from 16 embryos, injected with gAdprhl1-e6-1
L-homeolog
Expected sequence
exon 6
-e6-1
PAM
Observed sequences - WT
Observed sequences - deletion mutants
Observed sequences - insertion mutants
Non-injected sibling control sequence
Number of sequences
Genotype score
Total = 249
