# Supplementary material for: Defective heart chamber growth and myofibrillogenesis after knockout of adprhl1 gene function by targeted disruption of the ancestral catalytic active site
Source: PLoS One. 2020 Jul 29;15(7):e0235433. doi: 10.1371/journal.pone.0235433 (PMC7390403; doi:10.1371/journal.pone.0235433)

S15.

Range of ventricle phenotype severities observed after mutation of *adprhl1* exon 6

gRNA: gAdprhl1-e6-1

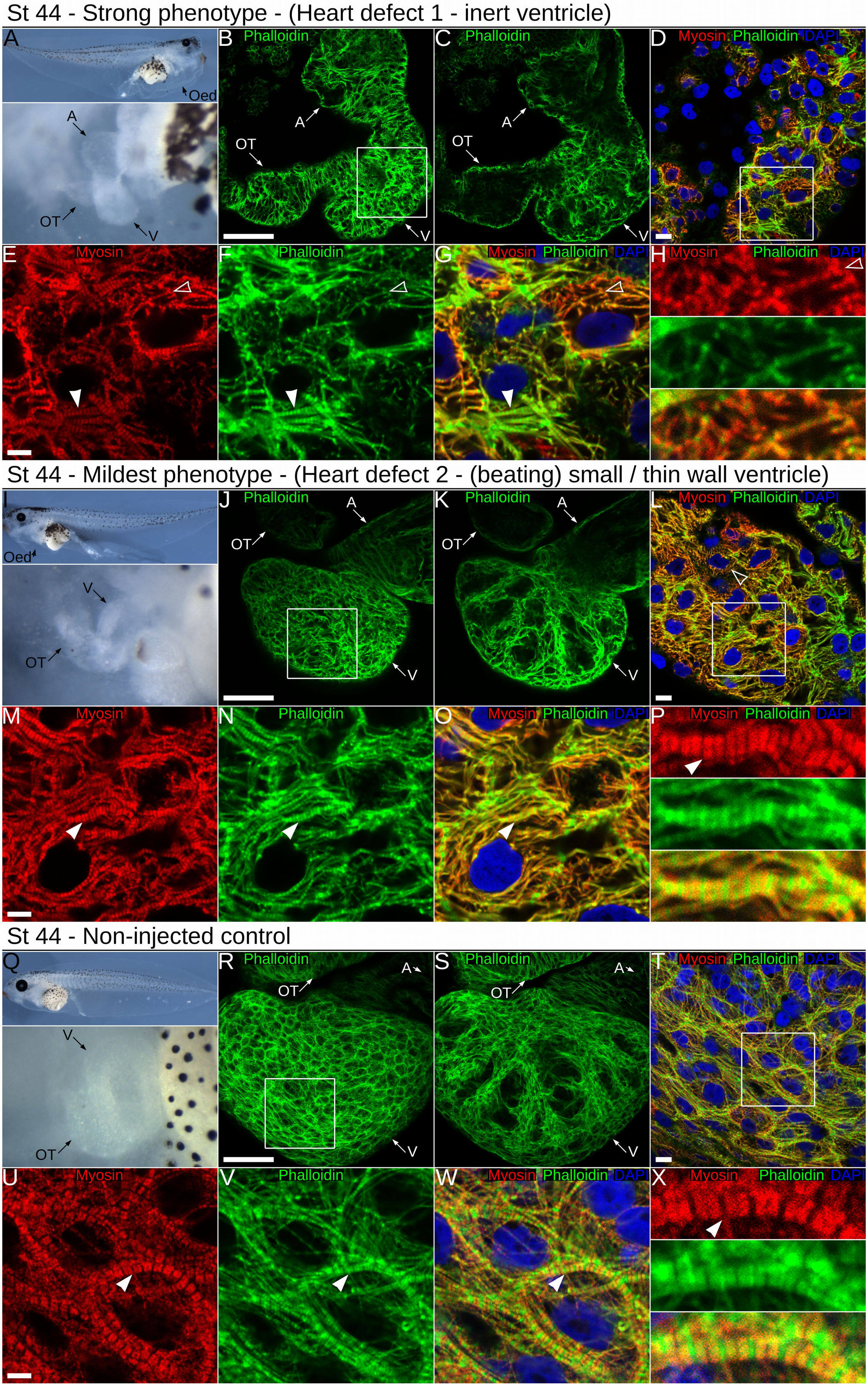

Supplement: S15 Fig — After adprhl1 mutation, tadpole heart phenotype was assessed at stage 44 and animals with aberrant morphologies were divided into two severity classes based on whether the ventricle was able to contract or not. The two examples here represent extremes from the range of hearts considered abnormal, from the most severely affected inert ventricle (A-H) to the mildest malformation observed in the beating ventricle class (I-P). It should be noted that most of the abnormal hearts were of an intermediate severity, were assigned to the beating group and resembled the earlier stage 40–42 examples shown in the principal figure (Fig 7), particularly with regard to the mosaicism found amongst the ventricular cardiomyocytes. A: Strong phenotype. Cardiac oedema of a stage 44 tadpole after injection of the gAdprhl1-e6-1 gRNA plus Cas9. Right-lateral view of tadpole and left detail of small inert heart presented. Aside from the heart, there are no other discernible defects. Axial structures are straight and gut looping has commenced. B, C: Fluorescence images of the dissected heart placed with anterior-left surface uppermost showing phalloidin actin filament stain (green) scanned at the level of the ventricle myocardial wall (B) and a slice through the lumen (C) located 10 μm deeper. Ventricle growth has completely failed and no trabeculae ridges have formed at the inner surface of the chamber. D-G: Abnormal cardiomyocytes (D) with merged signals for phalloidin actin (green), anti-myosin (red) and DAPI nuclei (blue) from the region of ventricle wall framed by the white square (B). The white square (D) in turn marks the further magnified images (E-G) that show separate myosin and actin signals in addition to the channel merge. H: Muscle filaments inside a single cardiomyocyte identified by the open arrowhead (E-H). The cardiomyocytes vary with regard to the composition of their myofibril structure. Many retain a round shape and contain clusters of short, thin muscle filaments. These [file pone.0235433.s015.pdf]
