# Supplementary material for: Defective heart chamber growth and myofibrillogenesis after knockout of adprhl1 gene function by targeted disruption of the ancestral catalytic active site
Source: PLoS One. 2020 Jul 29;15(7):e0235433. doi: 10.1371/journal.pone.0235433 (PMC7390403; doi:10.1371/journal.pone.0235433)
Supplement: S1 Data — (DOCX) [file pone.0235433.s019.docx]

5. Supplementary Methods.

**5.1. Background information on CRISPR/Cas9 optimization in *Xenopus* embryos.**

In aquatic vertebrate species like *Xenopus*, several approaches to targeted gene mutation are possible. The quickest is to supply Cas9 and gRNA reagents to newly fertilized embryos in sufficient quantity to cleave all DNA copies of the selected gene and instantly reveal mutant phenotypes as the injected embryos develop. A more traditional method would be to induce DSBs in a portion of the alleles, get germline transmission of a mutation and breed subsequent generations to obtain homozygous mutant animals. Gene mutations have been produced using both approaches in *X. tropicalis*, while the longer maturation time of *X. laevis* frogs and larger pseudo-tetraploid genome mean only the rapid, transient gene knockout experiments are practical. Nonetheless, the large and robust *X. laevis* embryo offers the experimental advantage of a longer time window for Cas9 to act prior to DNA synthesis occurring at the one-cell stage and has only four copies of a given gene that would require disrupting. Knockout phenotypes of well characterised genes have been obtained at higher frequency, and in greater number, using G0-generation *X. laevis* rather than *X. tropicalis* [24].

Initial studies in *Xenopus* supplied the endonuclease in the form of a Cas9 RNA. The RNAs sourced from different labs had distinct nucleotide sequences, some with codons optimised for use in mammalian species and the addition of one or two nuclear localization signals. There were obvious differences in efficacy of the Cas9 RNAs in *Xenopus*, with those utilizing RNAs with low *in situ* activity forced to compensate by increasing the mass of RNA injected (to unusual >2 ng quantities) and consequently experiencing problems with embryo toxicity [56]. Injection of Cas9 protein has taken over from RNA [57]. It allows a pre-incubation step to associate gRNA with the Cas9 that can facilitate instant activity once injected. With protein injection rather than Cas9 RNA, there is also lower risk of DNA damage from prolonged nuclease action beyond the first few cell divisions.

**5.2. Design of synthetic guide-RNAs.**

Selection of the *X. laevis adprhl1* gRNA sequences that were used in partnership with *S. pyogenes* Cas9 followed several design criteria. The entire exonic coding sequences of both S- and L-homeologous alleles were assessed. Where sequence differences existed between S- and L-alleles, separate gRNAs were prepared for each homeolog and then mixed prior to use. Only four sequences matched a GGN_18_-(NGG) consensus that would enable *in vitro* transcription of gRNA from a plasmid template. Some additional gRNAs were selected (representing each *adprhl1* exon) that contained longer 21-22 nucleotides of gene-specific sequence. Ultimately, adopting PCR-based transcription templates gave greater freedom of gRNA sequence choice. It was possible to select GN_19_-(NGG) sequences for gRNAs that target specific regions of interest within the *adprhl1* locus, notably those that encode the ancestral active site of the protein.

Current computational tools used to predict off-target activity of gRNAs have difficulty advancing beyond sequence alignment complementarity towards identifying those potential off-targets that are likely to be cleaved *in vivo* [Reviewed 25]. Here, all gRNA sequences were assessed for hybridization at other positions using Xenbase (www.xenbase.org/, RRID:SCR_003280) [26], the JGI *X. laevis* genome v9.2 [27] and Cas-Offinder [28], which considered the potential for both sequence mismatches and bulges. Results for potential off-targeting by the most active gAdprhl1-e6-1 gRNA to other gene coding sequences with up to two changes tolerated are listed below. None of the additional genes would be anticipated to contribute to a heart phenotype if mutated and more importantly, none of the potential interactions align with both of the homeologous alleles present in *X. laevis*.

Gene Exon Expression Mismatch DNA bulge RNA bulge.

nefm.L 3 Hindbrain, spinal cord 0 0 2 bases near 5’-.

stac3.S 2 Skeletal muscle only 1 (within seed)0 1 base near 5’-.

spryd3.S 10 brain 1 (within seed)0 1 base near 5’-.

Xelaev18028958m.g Not determined (ND) 1 (within seed)0 1 base near 5’-.

Xelaev18038829m.g ND 1 (within seed)0 1 base near 5’-.

Xelaev18046191m.g ND 1 (within seed)0 1 base near 5’-.

LOC100490289-like.L ND 1 (within seed)0 1 base near 5’-.

LOC100497528.S ND 1 (within seed)0 1 base near 5’-.

**5.3. Transcription of guide-RNAs and Cas9-encoding RNAs.**

Two different methods were employed for synthesis of gRNAs. Where the gene-specific element of a gRNA began with a 5’-GG sequence, a plasmid template intermediate was produced. The pUC57-Simple-gRNA backbone plasmid (Addgene:51306, Yonglong Chen) [30] facilitated cloning of annealed complementary oligonucleotides with 4 base 5’-overhangs. Oligo-1: 5’-TAGGN_18_ (transcription start site underlined). Oligo-2: 5’-AAACN^rev comp^_18_. Plasmid DNA was linearized with *DraI* and used as template in a MEGAshortscript^TM^ T7 transcription (Ambion). Transcribed gRNA was purified by ethanol precipitation and resuspended in 50 µl water.

Where the gRNA contained only a single 5’-G, a template DNA was prepared directly by PCR [Adapted from 57]. Long overlapping complementary oligonucleotides supplied all the necessary components: T7 RNA polymerase promoter, gene-specific and sgRNA sequences.

Oligo-1: 5’-GAAATTAATACGACTCACTATAGN_19_GTTTTAGAGCTAGAAATAGC.

Oligo-2: 5’-AAAAGCACCGACTCGGTGCCACTTTTTCAAGTTGATAACGGACTAGCCTTATTTTAACTTGCTATTTCTAGCTCTAAAAC (this oligo universal to all templates).

The two oligonucleotides were annealed and a PCR-extension reaction performed using Platinum SuperFi DNA polymerase (Invitrogen), 50 µl reaction, 0.2 µM oligos, 25 cycles of 98 °C 10 sec, 55 °C 10 sec, 72 °C 10 sec. Template DNA was eluted through a QIAquick PCR purification column (QIAGEN) and added to a MEGAshortscript^TM^ T7 transcription (Ambion). The transcription incubation time was increased to 5-7 hours to compensate for the suboptimal T7 promoter sequence.

Four different Cas9-encoding RNAs were tested for activity in *tyrosinase* gene knockout experiments, although Cas9 protein ultimately proved most effective in this assay (EnGen^®^ Spy Cas9 NLS, NEB: M0646M). Two Cas9 RNA variants were sourced from Addgene plasmids, pCS2-Cas9 (Addgene:47322, Alex Schier) [58] and pCS2+hSpCas9 (Addgene:51815, Masato Kinoshita) [59]. The Cas9-2A-OFP coding sequence (GeneArt:A21174) was purified using *NcoI-PmeI* sites and cloned into a modified pCS2. An OFP-2A-hSpCas9 sequence was prepared by cloning a synthesized OFP-2A- Strings DNA fragment (GeneArt) into pCS2+hSpCas9 using *BamHI-NcoI* sites. All plasmids were linearized with *NotI* and transcribed using mMessage mMachine^TM^ SP6 reactions (Ambion). RNA was purified by LiCl precipitation and resuspended in 50 µl water.

**5.4. *Tyrosinase* gRNA sequences.**

Two gRNAs were previously designed against both *X. laevis tyrosinase* homeologous alleles [24]. Their gene-specific sequences correspond to the *tyr* DNA listed below.

*tyra*-T: 5’-GGGTCGATGATAGAGAGGAC (direction: →, PAM: TGG).

*tyrb*-T: 5’-GGCCCGTAGCAGAGCTGGTG (direction: ←, PAM: AGG).

**5.5. *Adprhl1* gRNA sequences.**

The *adprhl1* sequences used to design the principal gRNAs used in the study are listed below. Mismatched bases of control gRNAs are coloured red.

gAdprhl1-e3-1S: 5’-GGGATGAGATACTGGAAACC (direction: →, PAM: AGG).

gAdprhl1-e3-1L: 5’-GGGATGAAATACTGGAAACC (direction: →, PAM: AGG).

gAdprhl1-e4-1: 5’-GGGCCGTGCACAGGGACCCA (direction: ←, PAM: AGG).

gAdprhl1-e6-1: 5’-GAGGGAAGAGGGGGAAGAAG (direction: →, PAM: AGG).

gAdprhl1-e6-1Mis1: 5’-GAGGGAAGAGGGGGAACAAG (direction: →, PAM: AGG).

gAdprhl1-e6-1Mis2: 5’-GAGGGAAGAGGGGGAACTAG (direction: →, PAM: AGG).

Additional *adprhl1* gRNAs were also tested for activity, some with longer 21-22 nucleotides of gene-specific sequence. Mismatched 5’-bases added to enable efficient T7-transcription are coloured blue.

gAdprhl1-e1-1: 5’-GGCAAGAAGAGCTAAAACAACT (direction: →, PAM: TGG).

gAdprhl1-e1-2: 5’-GGTGAGACTAGTGATTCCGCTG (direction: ←, PAM: TGG).

gAdprhl1-e2-1: 5’-GGCACACCCCATTCAATGAAAA (direction: →, PAM: AGG).

gAdprhl1-i2-1L: 5’-GGAAAGCACTTAGTGACCAG (direction: ←, PAM: TGG).

gAdprhl1-i2-2L: 5’-GGGAATGTAATAAAAATTAG (direction: ←, PAM: TGG).

gAdprhl1-e4-2S: 5’-GGAGAAACCACTTGCTCAGTG (direction: →, PAM: GGG).

gAdprhl1-e4-2L: 5’-GGAAAAACCACTTGTTCAGTG (direction: →, PAM: GGG).

gAdprhl1-e4-3S: 5’-GGAAGAAGACTATACGGCACA (direction: →, PAM: TGG).

gAdprhl1-e4-3L: 5’-GGAAGAAGACTATTCGGCACA (direction: →, PAM: TGG).

gAdprhl1-e5-1S: 5’-GGTTTTATTTTGAAGCCAAG (direction: →, PAM: TGG).

gAdprhl1-e5-1L: 5’-GGTTTTATTTTGAAACCAAG (direction: →, PAM: TGG).

gAdprhl1-e7-1S: 5’-GCAGGAGAAGGTGGTGCCAC (direction: →, PAM: TGG).

gAdprhl1-e7-1L: 5’-GCAGGAGAAGGTGGTGCTAC (direction: →, PAM: TGG).

gAdprhl1-e7-2S: 5’-GGTGTCTTTATGGATTGCTCTA (direction: →, PAM: TGG).

gAdprhl1-e7-2L: 5’-GGTGTCTGTATGGATTGCTCTA (direction: →, PAM: TGG).

Note -e5-1 (S and L) fits the optimal gRNA consensus but gave consistently poor synthesis yields due to an adverse T-repeat sequence and could not be assessed by injection into embryos.

**5.6. Cas9 and gRNA injection into *Xenopus laevis* embryos.**

Preliminary experiments tested the activity of different Cas9 RNAs by co-injecting them with the two gRNAs that target *tyrosinase*. A mixture containing a Cas9 RNA (125 pg/nl) and the two gRNAs (both at 125 pg/nl) was prepared and 4 nl injected into one-cell stage embryos, giving a final 500 pg mass of each reagent. Injections were directed towards the animal pole of the embryo (uppermost third). Injection of embryos continued from 35 until 60 minutes post-fertilization. They were incubated at room temperature (22 °C) until 90 mpf, then transferred to 17 °C. Culture media used for injection and first 24 hours incubation was 0.1xNAM, 0.5 % Ficoll^®^-400, 20 µg/ml gentamycin. Thereafter, 0.1xNAM was used.

Greater efficiency of *tyrosinase* and *adprhl1* gene knockout was achieved using a commercial Cas9 protein preparation, EnGen^®^ Spy Cas9 NLS (NEB: M0646M). In this case, gRNAs were preloaded onto Cas9 protein using a mixture assembled in the following order: 1 µl 1.3 M KCl (302 mM final), 2 µl gRNA (<1 µg of a single gRNA, so <233 pg/nl final) and 1.3 µl EnGen Cas9 (26 pmol). The mixture was incubated at 37 °C for 10 minutes, immediately prior to injection. Injection of 4 nl mixture and subsequent embryo culture was as before. The method was adapted from Burger *et al* [29]. For *tyrosinase* knockout, the phenotype classes used to define the extent of pigmentation-loss are described in Supplementary S7 and follow those of Guo *et al* [30].

For *adprhl1* knockout, embryos that gastrulated normally were allowed to develop to tadpole stage 44. Their external morphology was recorded each day and the appearance of their heart closely monitored. Tadpoles were assigned to one of four distinct phenotype classes, which differed slightly from those used to assess morpholino injection (see Supplementary Methods 5.12):

Heart defect 1 - inert ventricle. As per MO study.

Heart defect 2 - thin wall ventricle. In tadpoles showing a cardiac oedema that produced a beating heart, the ventricle was frequently thin-walled and became increasingly dilated by stage 44. This was especially true for embryos that received the exon 6 -e6-1 gRNA.

Other malformations. Any non-cardiac developmental defect visible externally by stage 44, however subtle.

Normal (heart) morphology. Perfect development through to stage 44.

There was no need to define a separate phenotype class for tail malformations as they did not occur in these CRISPR/Cas9 experiments (in contrast to Adprhl1-ATGMO1 morpholino injections). Control gRNA experiments were performed on the same day as an active -e6-1 gRNA injection to make use of identical batches of Cas9, testis, eggs and culture media.

**5.7. Sanger sequence analysis of mosaic *adprhl1* exon 3, 4 and 6 mutations in G0-generation *X. laevis*.**

DNA was extracted from individual tadpoles. Frozen tissue was mixed with 200 µl 50 mM Tris pH8.8, 1 mM EDTA, 0.5% Tween20 containing freshly added 600 µg/ml proteinase K and incubated at 55 °C for 20 hours. PCR amplification of *adprhl1* genomic sequences used Platinum SuperFi DNA polymerase (Invitrogen) and 1 µl tadpole extract per 30 µl reaction. Plasmid clone isolates of amplicon DNA were prepared using a Zero Blunt^TM^ TOPO^TM^ PCR cloning kit (K287520, Invitrogen) and their inserts were sequenced. Sanger sequencing allowed study of 2 kbp amplicons so that the presence of larger deletions and insertions could be detected.

PCR primers for *adprhl1* genotyping.

Exon 3 genotyping. (position, direction).

p2483: 5’-TGCAAAGAGGGTTCTTTAGGGAAG (intron 2, →).

p2460: 5’-GTCATTCTCCCACTTTCAATGCTGAC (exon 3, ←).

p2555: 5’-TTGAAACCAGATAACTACCTG (exon 2, →).

p2556: 5’-TCTCCCACTTTCAATGCTGAC (exon 3, ←).

p2566: 5’-AGCCTCCCCGTATTCTCTAAG (intron 2, →).

p2569: 5’-GAAATATTTATAGATTTTCATAAGGTGG (intron 3, ←).

PCR fragment sizes.

p2483+p2460, S-homeolog=161 bp, L=161 bp.

p2555+p2556, S=1944 bp , L=1326 bp.

p2566+p2569, S=426 bp.

Exon 4 genotyping. (position, direction).

p2515: 5’-TCTTCTGCCATGAGACAAGGT (intron 3, →).

p2517: 5’-TCTTCCAGGTAAAACTGCCAC (exon 5, ←).

p2541: 5’-GAGCAATACCCAGAGTTTCTT (intron 3, →).

p2542: 5’-GAGCAATACCCAGAGGTGCTT (intron 3, →).

PCR fragment sizes.

p2515+p2517, S=876 bp.

p2541+p2517, S=893 bp.

p2542+p2517, L=944 bp.

Exon 6 genotyping. (position, direction).

p2560: 5’-AGTTTTACCTGGAAGAAAGAG (exon 5, →).

p2648: 5’-GTGAGCTTATCTTTACAAGTATC (intron 6, ←).

p2649: 5’-TAAAGGGACACTTTCTTATCCAG (intron 6, ←).

p2514: 5’-TCATCTCAAGCTGCTGGTATA (exon 7, ←).

p2657: 5’-GGCTGGTCATGTGGCATGGTCA (intron 6, ←).

PCR fragment sizes.

p2560+p2648, S=413 bp, L=404 bp.

p2560+p2649, S=604 bp, (L=592 bp).

p2560+p2514, S=2133 bp, L=1774 bp.

p2560+p2657, L=638 bp.

Each mutated sequence was assigned a genotype score (a number code) according to the size of the amino acid lesion that it encoded. This classification of mosaic mutations found within an individual tadpole was then compared against its cardiac morphology. Some assumptions were made for sequence deletions that disrupted exon splice junctions. Where a splice acceptor site was lost, it was assumed the exon was skipped. Where a splice donor sequence was removed, it was assumed the following intron was inappropriately retained.

Genotype score codes.

01: Inactive mutant. Frame-shift-stop or nonsense mutation.

02: In-frame mutant causing more than 20 amino acid changes.

03: In-frame mutant causing between 11-20 amino acid changes.

04: In-frame mutant causing 6-10 amino acid changes.

05: In-frame mutant causing 1-5 amino acid changes.

06: Normal amino acid sequence.

**5.8. Amplicon-EZ (NGS) sequence analysis of mosaic *adprhl1* exon 6 mutations.**

The Amplicon-EZ next generation sequencing service (Genewiz) was used as a cost-effective method to obtain deeper coverage of the mosaic exon 6 mutations from individual tadpoles, providing 50,000 reads per sample. Genomic PCRs obtained with primers p2560+p2648 were sequenced directly using Illumina^®^ technology. The Galaxy web platform (www.usegalaxy.eu) [31] provided the following tools to analyse the data. A Galaxy tutorial [60] was adapted for read mapping and subsequent mapped read filtering steps.

FastQC and MultiQC assessed sequence quality of the compressed fastq files.

Trimmomatic was employed to trim the paired-end reads and incorporated an initial Illuminaclip step to cut adaptor sequences.

Illuminaclip settings (default): Adapter sequences to use, TruSeq3 (paired-ended, for MiSeq and HiSeq); Maximum mismatch count which will still allow a full match to be performed, 2; How accurate the match between the two 'adapter ligated' reads must be for PE palindrome read alignment, 30; How accurate the match between any adapter etc. sequence must be against a read, 10; Minimum length of adapter that needs to be detected (PE specific/palindrome mode), 8; Always keep both reads (PE specific/palindrome mode)?, yes.

Three additional Trimmomatic operations were: Cut the specified number of bases from the start of the read (HEADCROP), 3; Cut bases off the end of a read, if below a threshold quality (TRAILING), 10; Drop reads below a specified length (MINLEN), 25.

For mapping the paired-end reads, a fasta file containing both control amplicons of the *adprhl1* S- and L-alleles (413 and 404 bp) was uploaded to supply reference sequences. The Map with BWA-MEM tool assembled the reads and was used to manually assign read group information, a read group identifier (ID) and read group sample name (SM) that was specific for each tadpole and its heart phenotype class.

Extra Map with BWA-MEM settings (default): Algorithm for constructing the BWT index, Auto. Let BWA decide the best algorithm to use; Platform/technology used to produce the reads (PL), ILLUMINA; Select analysis mode, 1. Simple Illumina mode; Other fields were left blank.

Post-processing of the mapped reads used the Filter BAM datasets on a variety of attributes tool. Three simple filters were applied: 1 mapQuality, Filter on read mapping quality (phred scale), >=1; 2 isMapped, Selected mapped reads, Yes; 3 isMateMapped, Select reads with mapped mate, Yes; Would you like to set rules? No.

The Integrative Genomics Viewer (IGV) [61] was used to visualise the mapped reads of the filtered BAM files. Within View/Preferences/Alignments, Downsampling was not selected so that all the sequence reads were displayed. For Alignment Track Options, Filter supplementary alignments was toggled to show/hide a small number of instances where a read was mapped to a second separate position on the reference sequence, in order to avoid double-counting within the total number assembled (a few reads that contained the largest deletions spanning the gRNA site had mapped separately to each side of their breakpoint).

The total number of assembled reads recorded for each tadpole counted only those mapped with reverse strand orientation as the -e6-1 gRNA sequence occurred towards the 3’- of the PCR product. Thus the previously filtered BAM files were returned as inputs to the same Galaxy Filter BAM datasets on a variety of attributes tool using the parameter: 1 isReverseStrand, Select reads in the reverse strand only, Yes; Would you like to set rules? No.

In order to examine whether any wild-type alleles persisted after -e6-1 gRNA mutation, a different filter was employed to refine the search process. The originally filtered BAM files were input to Filter BAM datasets on a variety of attributes, using the parameter: 1 tag, Filter on a particular tag, NM:<4; Would you like to set rules? No. Restricting the number of mismatches permitted to less than four reduced the number of reads to screen while still allowing through sequences containing some natural variations that were distant from the gRNA site. Once these remaining reads were displayed within IGV, we focussed on a 15 bp sequence that included the PAM (5’-GGGGGAAGAAGAGGA, S- PCR bases-263-277, L- 254-268). The Alignment Track Pop-up Menu was used to group and sort reads by their sequence at defined bases. A wild-type read was defined as having the normal translated peptide sequence ^269^GGRRG^273^.

**5.9. Western blot detection of Adprhl1 protein.**

Adprhl1 protein was detected using a rabbit antibody raised against an ADPRHL1 peptide (mouse ^248^DNYDAEERDKTYKKWSSE^265^, encoded by exons 5-6) [10]. The antibody is active against the *Xenopus*, mouse and human species orthologs. For protein extraction from embryonic *Xenopus* hearts, typically 100 hearts were dissected, pooled, snap frozen, homogenized in 120 µl RIPA buffer and boiled with an equal volume of 2x reducing protein sample buffer. Individual adult female mouse hearts were homogenized in 400 µl RIPA, the resulting slurry mixed with 400 µl sample buffer, then aliquots diluted a further three-fold with RIPA/sample buffer before SDS-PAGE.

**5.10. Immunocytochemistry of *Xenopus* hearts.**

Immunocytochemistry was performed on whole tadpoles and subsequently the hearts were dissected, mounted in 12 µl CyGEL Sustain (biostatus) and viewed using Zeiss LSM5-Pascal or LSM710 confocal microscopes. Confocal images of whole hearts captured 2 µm deep optical sections. Both the ventricle myocardial wall and also deeper trabecular layers were assessed by scanning different depths. Images of cardiomyocytes and myofibrils were 1 µm optical sections, at a depth 1-2 µm below the outer (apical) myocardial surface. Antibodies used were Adprhl1, Myosin A4.1025 (DSHB) and phospho-Histone H3(Ser10) 3H10 (Sigma), along with fluorescent dye-conjugated secondary antibodies (Jackson ImmunoResearch). Atto 633-conjugated phalloidin (Sigma) stained actin filaments. Dying cells were visualized with the ApopTag^®^ Red In Situ Apoptosis Detection kit (Sigma).

**5.11. *Adprhl1* morpholino sequences.**

Morpholino oligonucelotides (Gene-Tools) were previously designed to interfere with *X. laevis adprhl1* RNA-splicing [10] while new MOs aimed to inhibit the initiation of protein translation. For translation inhibition, MOs were synthesized that matched both S- and L-homeologous alleles and their activities were assessed both individually and mixed together. Furthermore, distinct MOs were designed to hybridize to every ATG-sequence within the first three exons that could initiate translation in the correct reading frame, in an attempt to identify the N-terminus of the 23 kDa Adprhl1 protein species.

For the RNA-splice interfering MOs, the advent of the improved JGI *X. laevis* genome v9.2 has revealed some sequence variability. Nonetheless, the two MOs remain valid for targeting both S- and L-alleles (Supplementary S5. Note, previous RT-PCR analysis would have successfully detected both homeologous alleles [10]). For Adprhl1-e2i2MO, approximately half of the L-alleles sequenced are a perfect match to the MO while half contain two base mismatches (GenBank accessons GU188989, GU188990 have matching sequence, JGI genome v9.2 J-strain allele has mismatches at MO bases 6 and 15). For Adprhl1-i2e3MO, half the L-alleles sequenced match the MO and half contain a single mismatch (J-strain allele has the matching sequence, GU188989 has a mismatch at MO base 25).

Sequences.

Adprhl1-ATGMO1(S1a): 5’-CCTTAAACTTCTCCATAGCAAGGGC-3’.

Adprhl1-ATGMO1(S1b): 5’-TGCAGCCTTAAACTTCTCCATAGCA-3’.

Adprhl1-ATGMO1(L1): 5’-CATCGCAGCCTTAAACTTCTCCATG-3’.

Adprhl1-ATGMO2(L2): 5’-GGATCTTTACTCCTGAAACACACAT-3’.

Adprhl1-ATGMO3(S,L3): 5’-ATTAGAGTATTGTTACTCACTGGCC-3’.

Adprhl1-ATGMO4(S4): 5’-GTTTTACCATGTCACGGTACAGGTC-3’.

Adprhl1-ATGMO4(L4): 5’-GTTTCACCATGTCACGATACAGGTC-3’.

Adprhl1-ATGMO5(S5): 5’-AGTATCTCATCCCAATACACATGGC-3’.

Adprhl1-ATGMO5(L5): 5’-AGTATTTCATCCCAATACACATGGC-3’.

Adprhl1-ATGMO6(S6): 5’-GATGGTTATGAGTCATTCTCCCACT-3’.

Adprhl1-ATGMO6(L6): 5’-GATGGTTATGCGTCATTCTCCCACT-3’.

Adprhl1-ATGMO6Mis: 5’-GATAGTTATAAGTAATTCTACCAAT-3’.

Adprhl1-e2i2MO: 5’-AGGCTCAGCATCTTACAAACCTTTT-3’.

Adprhl1-e2i2MOMis: 5’-AAGCTAAGCATATTAAAAACATTTT-3’.

Adprhl1-i2e3MO: 5’-ACCTAAGAAACAACTAGAGTCACTG-3’.

Adprhl1-i2e3MOMis: 5’-AACTAAAAAACAAATAGAATCAATG-3’.

Aside from the described sequence similarity between ATGMO6 and the ADP-ribosylhydrolase gene family, BLAST searches did not indicate any potential MO hybridization to other *Xenopus* mRNAs or genomic regions that could cause off-target activity. Nonetheless, all potential interactions are listed below.

MO Aligns with Complementarity Strand (MO seq).

L2 *Xelaev18011653m*, exon 2 20/25 sense (+) strand.

L2 *ago2.L*, intron 1 20 + strand.

S6 *LOC108699836*, intron 14 20 - strand.

e2i2MOMis *irp10.L*, exon 5 21 - strand 3'-UTR.

i2e3MOMis *gda.L*, intron 13 20 - strand.

**5.12. Morpholino injection into *Xenopus laevis* embryos.**

When the Adprhl1-e2i2MO morpholino was combined with stable transgenic *adprhl1* over-expression lines, embryos were injected into both dorsal blastomeres at the four-cell stage [10]. In all other experiments, MOs were injected at the one-cell stage in order to provide a better comparison with the CRISPR/Cas9 *adprhl1* gene knockout. The RNA-splice interfering MOs gave a slightly reduced effectiveness with one-cell stage injection because it results in a lower relative concentration within heart forming tissue. Two masses of MO were assessed, 32 ng and 16 ng. All embryos that gastrulated normally were allowed to develop to tadpole stage 44. Their external morphology was recorded each day.

Tadpoles were assigned to one of five distinct phenotype classes:

Heart defect 1 - inert ventricle. First visible sign of aberrant morphology at stage 40-41, with developing cardiac oedema. Heart remains small, string-like. The cardiac pacemaker initiates pulsing contractions but the small ventricle region remains inert.

Heart defect 2 - (beating) ventricle malformations. A broader class of cardiac phenotypes. First sign of aberrant morphology again at stage 40-41, with developing cardiac oedema. The forming ventricle does propagate a heart beat but is malformed. The most common malformation is a small ventricle. Other cardiac malformations observed include a thin-walled ventricle, becoming increasingly dilated by stage 44, or a ventricle with incorrect left-right and antero-posterior orientation.

Tail defect. First sign of aberrant morphology at stage 32, showing a twist in the trunk region and a loss of tail outgrowth. Tail defect becoming more extreme by stage 44. Heart malformation frequently observed but difficult to ascertain whether a primary defect or a consequence of failed development elsewhere.

Other malformations. This class mostly found with MO-L6. Normal gastrulation but subsequently develop severe malformations by stage 24.

Normal morphology. Perfect development through to stage 44.

**5.13. Binary transgene system for cardiac Adprhl1 expression.**

The design of transgenes for cardiac over-expression of Adprhl1 proteins has been described previously [10]. The full name of the principal transgenes used here are: *Tg[myl7:Gal4, γCrys:eCFP]*, Tg[UAS:human^1-52^-Xenopus^53-354^ adprhl1, γCrys:DsRed1] and Tg[UAS:Xenopus adprhl1(silent 1-282bp), γCrys:DsRed1]. Additional responder transgenic lines featured as controls for western blot detection of recombinant proteins. The *X. laevis* *adprhl1* cDNA was from IMAGE:4409193 and corresponded to the S-homeologous allele sequence. The 5’-cDNA of human *ADPRHL1* was from IMAGE:5299214.

**5.14. Binary transgene system for controllable genetic cell ablation.**

The *Tg[UAS:M2(H37A), γCrys:DsRed1]* line utilized the toxic mutant version (H37A) DNA of the *influenza* virus ion channel protein M2 [55]. The plasmid clone was produced exactly as for *adprhl1* responder transgenes.

**5.15. Hybridization probes.**

The *X. laevis adprh* S-allele cDNA used to synthesize an *in situ* hybridization probe was from IMAGE:6953995. Detection of *adprhl1* used a 789 base S-allele probe from coding cDNA 277-1065 bp [10], or a 1043 base L-allele probe from coding cDNA 1-1043 bp. Probe sequences for exons 1 to 7 were produced from the S-allele cDNA. The predicted L-allele exon 8 probes were obtained from genomic DNA. The exon 8 L-p1 product was identical to the JGI genome v9.2 while the L-p2 sequence had a small number of nucleotide changes and 36 bp of insertions (1376 bp versus predicted 1340 bp).

S-allele PCR primers for exon amplification. (position, direction).

p2509: 5’-ATGGAGAAGTTTAAGGCTGCA (exon 1, →).

p2621: 5’-CTGAGACTAGTGATTCCGCTG (exon 1, ←).

p2622: 5’-ATTACTGGAGCATAGAAGACC (exon 2, →).

p2623: 5’-CTTTTTCATTGAATGGGGTGT (exon 2, ←).

p2624: 5’-GTTCAGGATTTGGAGCTGCA (exon 3, →).

p2625: 5’-CTGTTGGATGGTTATGAGTCA (exon 3, ←).

p2626: 5’-GATTCCTTGGGTCCCTGTGC (exon 4, →).

p2627: 5’-CTGCCATGTGCCGTATAGTC (exon 4, ←).

p2628: 5’-AATATCAGGAACACTGGTTTTATTTTG (exon 5, →).

p2629: 5’-CTTGTCTCTTTCTTCCGCAT (exon 5, ←).

p2630: 5’-GCATACAAGACATGGAGCTC (exon 6, →).

p2631: 5’-CTCCATGGAACATTGCTCTG (exon 6, ←).

p2632: 5’-GAGAAGGTGGTGCCACTGGG (exon 7, →).

p2633: 5’-TTACTTTTCCATTGTAGCTAAGTG (exon 7, ←).

L-allele PCR primers for probe production. (position, direction).

p1855: 5’-ATGGAGAAGTTTAAGGCTGC (exon 1, →).

p1856: 5’-TGATAGAGCTTCTCGCCAAGG (exon 7, ←).

p2606: 5’-GTCAGTGCTTCATGGTGGTA (exon 8 L-p1, →).

p2610: 5’-AAGACTGGCCTGTTCTGCTA (exon 8 L-p1, ←).

p2608: 5’-GTTGTTAGACACCCCAACAA (exon 8 L-p2, →).

p2613: 5’-CACTTGCTGTAATCACTAGAGG (exon 8 L-p2, ←).

**5.16. Animals.**

Transgenic *Xenopus* and *Adprhl1^em1(IMPC)H^* allele (*em1*) mice were maintained at The Francis Crick Institute, according to the Home Office UK Animals (Scientific Procedures) Act 1986 under the Project Licence of Timothy Mohun. All *Xenopus* embryos were culled before reaching protected status (at or prior to developmental stage 44). Reversible anaesthesia of *Xenopus* embryos used 0.015% ethyl 3-aminobenzoate methanesulphonate (MS-222) in buffered 0.1xNAM. Terminal anaesthesia used 0.4% MS-222. Mice were bred, born and weaned at room temperature and maintained on a 12-hour light-dark cycle. Mice were housed in groups of four and fed a standard diet (2018s, Envigo). Mice were anaesthetised with 1.5–2.0% isoflurane in 2 L/min oxygen and euthanized by isoflurane. Mice were handled by experienced experimenters to minimize any eventual distress.

PCR primers for mouse *Adprhl1 em1* genotyping.

(position, direction).

p2570: 5’-CCGTGCATGTGGAGAGAGTT (intron 2, →).

p2579: 5’-TGGCTTCCAGTACCGCATTC (exon 3, ←).

p2582: 5’-GGTGTACACACGGGAGACTT (intron 2, →).

p2583: 5’-AGGCGGATTAATTCCTCTTC (intron 4, ←).

PCR fragment sizes.

p2570+p2579, WT=493 bp, *em1*=no product.

p2582+p2583, WT=1438 bp, *em1*=351 bp.

Supplementary References.

Additional works cited in the Supplementary Figure Legends or Supplementary Methods that do not feature elsewhere in the manuscript.

55. Smith SJ, Kotecha S, Towers N, Mohun TJ. Targeted cell-ablation in Xenopus embryos using the conditional, toxic viral protein M2(H37A). Dev Dyn. 2007;236: 2159–2171. doi:10.1002/dvdy.21233

56. Bhattacharya D, Marfo CA, Li D, Lane M, Khokha MK. CRISPR/Cas9: An inexpensive, efficient loss of function tool to screen human disease genes in Xenopus. Dev Biol. 2015;408: 196–204. doi:10.1016/j.ydbio.2015.11.003

57. Naert T, Colpaert R, Van Nieuwenhuysen T, Dimitrakopoulou D, Leoen J, Haustraete J, et al. CRISPR/Cas9 mediated knockout of rb1 and rbl1 leads to rapid and penetrant retinoblastoma development in Xenopus tropicalis. Sci Rep. 2016;6: 35264. doi:10.1038/srep35264

58. Gagnon JA, Valen E, Thyme SB, Huang P, Akhmetova L, Ahkmetova L, et al. Efficient mutagenesis by Cas9 protein-mediated oligonucleotide insertion and large-scale assessment of single-guide RNAs. PLoS ONE. 2014;9: e98186. doi:10.1371/journal.pone.0098186

59. Ansai S, Kinoshita M. Targeted mutagenesis using CRISPR/Cas system in medaka. Biol Open. 2014;3: 362–371. doi:10.1242/bio.20148177

60. Maier W. Identification of somatic and germline variants from tumor and normal sample pairs (Galaxy Training Materials). 17 Mar 2020. Available: https://galaxyproject.github.io/training-material/topics/variant-analysis/tutorials/somatic-variants/tutorial.html

61. Robinson JT, Thorvaldsdóttir H, Winckler W, Guttman M, Lander ES, Getz G, et al. Integrative genomics viewer. Nat Biotechnol. 2011;29: 24–26. doi:10.1038/nbt.1754
